# Transcriptional Divergence Underpinning Sexual Development in the Fungal Class Sordariomycetes

**DOI:** 10.1128/mbio.01100-22

**Published:** 2022-05-31

**Authors:** Wonyong Kim, Zheng Wang, Hyeonjae Kim, Kasey Pham, Yujia Tu, Jeffrey P. Townsend, Frances Trail

**Affiliations:** a Department of Plant Biology, Michigan State University, East Lansing, Michigan, USA; b Korean Lichen Research Institute, Sunchon National University, Suncheon, South Korea; c Department of Biostatistics, Yale School of Public Health, New Haven, Connecticut, USA; d Department of Ecology and Evolutionary Biology, Yale University, New Haven, Connecticut, USA; e Department of Mathematics and Computer Science, University of Strasbourg, Strasbourg, France; f Department of Plant, Soil and Microbial Sciences, Michigan State University, East Lansing, Michigan, USA; Universidade de Sao Paulo

**Keywords:** *Fusarium graminearum*, gene expression divergence, hypothetical proteins, perithecia development, single-copy orthologs

## Abstract

Gene expression divergence through evolutionary processes is thought to be important for achieving programmed development in multicellular organisms. To test this premise in filamentous fungi, we investigated transcriptional profiles of 3,942 single-copy orthologous genes (SCOGs) in five related sordariomycete species that have morphologically diverged in the formation of their flask-shaped perithecia. We compared expression of the SCOGs to inferred gene expression levels of the most recent common ancestor of the five species, ranking genes from their largest increases to smallest increases in expression during perithecial development in each of the five species. We found that a large proportion of the genes that exhibited evolved increases in gene expression were important for normal perithecial development in Fusarium graminearum. Many of these genes were previously uncharacterized, encoding hypothetical proteins without any known functional protein domains. Interestingly, the developmental stages during which aberrant knockout phenotypes appeared largely coincided with the elevated expression of the deleted genes. In addition, we identified novel genes that affected normal perithecial development in Magnaporthe oryzae and Neurospora crassa, which were functionally and transcriptionally diverged from the orthologous counterparts in F. graminearum. Furthermore, comparative analysis of developmental transcriptomes and phylostratigraphic analysis suggested that genes encoding hypothetical proteins are generally young and transcriptionally divergent between related species. This study provides tangible evidence of shifts in gene expression that led to acquisition of novel function of orthologous genes in each lineage and demonstrates that several genes with hypothetical function are crucial for shaping multicellular fruiting bodies.

## INTRODUCTION

Changes in gene expression underlie the interaction of developmental processes that drive morphological innovation in multicellular organisms (1–3). Although interspecies variations in expression of homologous genes have been linked to the evolution of complex phenotypes (4–9), emergent properties of these interactions leading to diverse morphologies are largely unstudied in filamentous fungi. Estimation of gene expression in the most recent common ancestors (MRCAs) provides a means of identifying significant shifts in gene expression that may have contributed to morphological diversification along divergent lineages. It has previously been hypothesized that genes whose expression increases substantially in one lineage during divergence of Fusarium and *Neurospora* species play key roles in the morphological innovations in these genera that represent two of the major evolutionary lineages in the fungal class Sordariomycetes (10). Genes exhibiting evolved differences in their expression levels were shown to be key developmental genes essential for sexual fruiting body development in Fusarium graminearum and Neurospora crassa (10). This study validated the use of inferred differences in gene expression between the MRCA and extant species to predict gene function in a developmental pathway that will manifest in the phenotype of gene knockout mutants.

Morphologies of ascomata, sexual fruiting bodies of the fungal phylum Ascomycota, has provided key taxonomic characters for classification until a recent “phylogenetic overhaul” that have largely eliminated the reliance on physical characteristics (11–14). Species featuring similar ascomata still tend to be grouped together in this reclassification, indicating that common ancestry is one basis of common ascomata morphology (11, 15). The Sordariomycetes is one of the largest taxonomic clades in the kingdom Fungi, which includes saprotrophic species and fungal pathogens that attack animals, insects, and plants (16, 17). Taxa of the Sordariomycetes are characterized by sexual reproduction with flask-shaped ascomata, called perithecia, which occupy a pivotal role in the life cycle of these fungi. Adaptive perithecial morphology is essential to survival under adverse environmental conditions, generation of novel genotypes by recombination, and local and long-distance dispersal of progeny in the form of meiotic spores called ascospores (16).

Genetic and molecular mechanisms of perithecial development, such as cellular signaling, gene silencing, and epigenetic controls, have been studied in model sordariomycete species F. graminearum, N. crassa, and Sordaria macrospora (18–25). Perithecia undergo morphogenic processes, sequentially developing five representative tissue types as they mature: ascogenous hyphae, perithecial walls, paraphyses (sterile hyphae), asci (sac-like structures harboring ascospores), and ascospores. Once environmental conditions become conducive to sexual development, layers of perithecial walls develop around the ascogenous hyphae, and then paraphyses fill the central cavity. The ascogenous system expands and forms a hymenium as the paraphyses senesce, becoming the predominant tissue and producing asci (26–29). These five tissues are specific for sexual development and their appearance provides morphological hallmarks that may be associated with shifts in gene expression relevant to their development and to the morphological diversity in sordariomycetes fungi.

**FIG 1:**
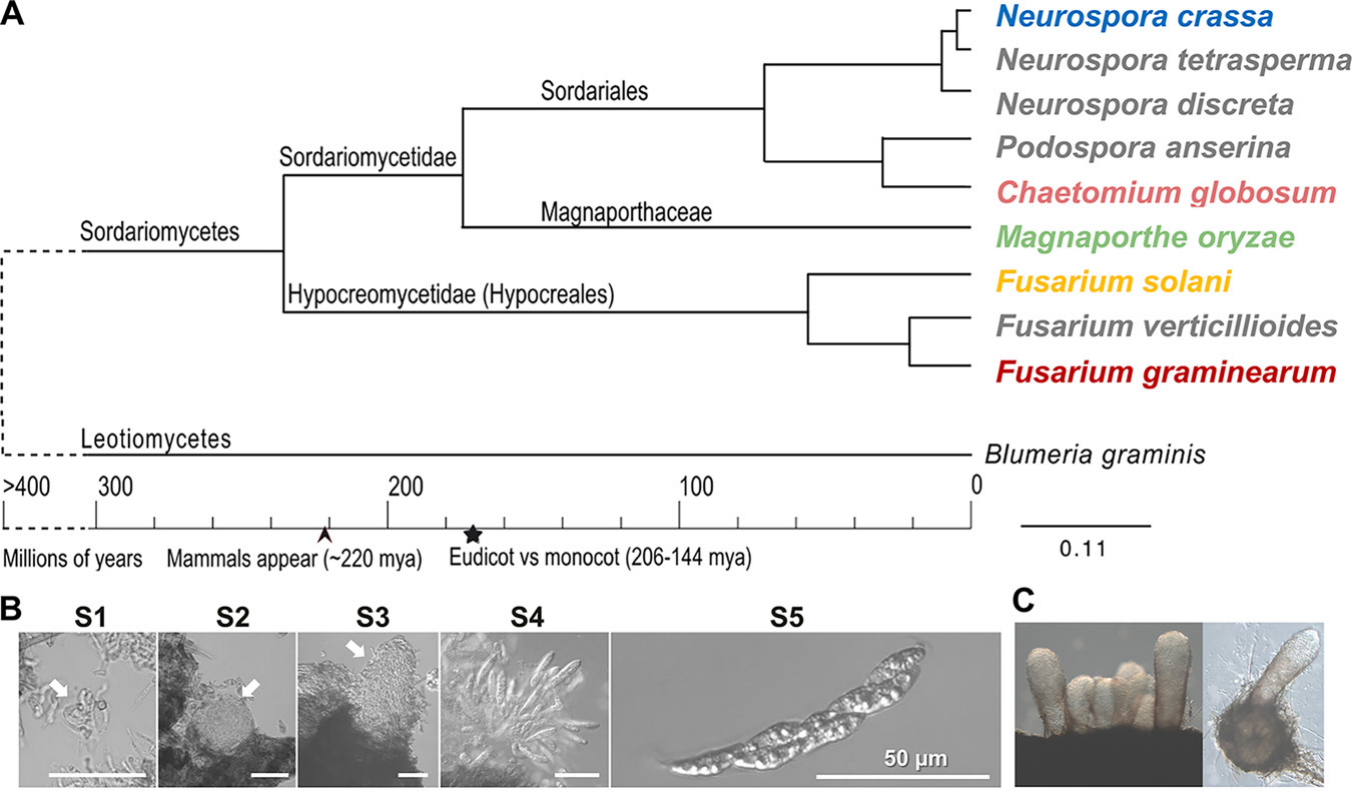
Phylogenetic relationships of studied fungal taxa and stages of perithecial development. (A) Maximum likelihood tree generated from large subunit (LSU) and small subunit (SSU) ribosomal DNA (rDNA) sequence data, revealing the relationships of major evolutionary lineages of the Sordariomycetes. Transcriptome profiles for perithecial development were obtained in five taxa labeled with unique colors. (B) Stages of perithecial development in Magnaporthe oryzae. Emergence of new tissues at the defined developmental stages (S1 to S5). Arrows in the S1, S2, and S3 panels indicate ascogonium, protoperithecium, and perithecial beak formation, respectively. Shown are developing asci released from a squashed perithecium (S4) and a deciduous ascus with eight ascospores (S5). (C) Cluster showing beaks of mature perithecia (left panel) and an isolated mature perithecium revealing embedded centrum (right panel).

Five fungal species, Chaetomium globosum, F. graminearum, Fusarium neocosmosporiellum, Magnaporthe oryzae, and N. crassa, from three deeply diverging lineages in the Sordariomycetes (Fig. 1A) were selected for study of perithecial development because they can be genetically manipulated, develop diverse morphologies of perithecia composed of the above-mentioned, well-characterized tissue types under laboratory conditions, and feature rich genomic resources that enable comparative and functional analyses (30–32). Fusarium graminearum is a devastating pathogen of wheat worldwide, and its sexual stage is essential to the disease cycle (33). The Fusarium solani species complex (FSSC) contains members that cause diseases in many agriculturally important crops, as well as saprotrophs and human pathogens (34). Fusarium neocosmosporiellum (syn. Neocosmopora vasinfecta) is an opportunistic human pathogen that belongs to an early-diverging clade of the FSSC (35, 36). Magnaporthe oryzae causes significant crop loss worldwide for rice, one of the most important food staples (26), and thus it has been the focus of intense study on host-pathogen interactions (37, 38). For more than 70 years, N. crassa has been a model system for studies in genetics and biochemistry, serving as the study organism for Beadle and Tatum’s “one gene, one enzyme” hypothesis (39). Chaetomium globosum is a cosmopolitan fungus that is a common contaminant on plant debris and often associated with terrestrial plants, marine algae, and lichens as endophytes (40–42). These five species all develop perithecia on carrot agar medium, a “common garden” environment that was established to create uniform growth conditions across species for comparative fungal transcriptome analysis (10).

Delineating the evolution of developmental programs at organismal levels requires systems-level approaches and measurable diversity of developmental morphology. The five selected species exhibit significant morphological diversity, facilitating improved insight into the evolving gene regulation associated with perithecial development. In this study, we applied comparative transcriptomic approaches to the inference of the systems biology underlying fungal fruiting body formation, revealing transcriptional divergence of orthologous genes in the five species. We performed targeted knockouts of genes estimated to have evolved increases in gene expression in F. graminearum, M. oryzae, and N. crassa compared to the MRCAs of the five species and identified a few novel developmental genes that play essential roles at different stages of perithecial development, many of which have evolved to have lineage- or species-specific function.

## RESULTS

### Transcriptome analysis during perithecial development.

Developmental sequences of perithecia can be defined by the sequential emergence of distinct tissue types at each stage (10, 26, 43). Stage 1 (S1) is hallmarked by development of ascogenous hyphae from the perithecium initials, stage 2 (S2) is distinguished by formation of perithecial walls surrounding the central ascogenous hyphae, stage 3 (S3) is characterized by the release of paraphysis tissues in squash mounts of perithecia and elongation of the perithecial beak in some fungi, including N. crassa and M. oryzae, stage 4 (S4) begins with development of asci in place of senescent paraphyses, and stage 5 (S5) is recognized by mature ascospores enclosed in asci. We obtained transcriptome data for *C. globosum*, F. graminearum, *F. neocosmosporiellum*, and N. crassa from our previous studies, in which all the fungal strains were grown on carrot agar medium used as a common garden environment, and their genome-wide gene expression levels were profiled at the five sexual developmental stages, including a vegetative stage right after sexual induction (S0) (10, 43–45). In this study, we also profiled the developmental transcriptome for M. oryzae, which belongs to a separate and divergent clade (Magnaporthaceae) from the other four species (Fig. 1A). Since M. oryzae is self-incompatible, we crossed two highly fertile strains with opposite mating types, 4091-5-8 (*MAT1-2*) and 4136-4-3 (*MAT1-1*), for sexual development (46). The 4091-5-8 strain was grown on carrot agar medium and fertilized with conidia from the 4136-4-3 strain. Mature perithecia of M. oryzae were formed in clusters embedded in heavily melanized hyphal matrices, and the transcriptome data for six developmental stages (S0 to S5) in M. oryzae were obtained by transcriptome sequencing (RNA-seq) (Fig. 1B). About 160 million RNA-seq reads were generated, with an average of 22 million reads mapped to the reference M. oryzae genome per developmental stage (see Table S1 in the supplemental material).

10.1128/mbio.01100-22.6 TABLE S1 Summary of HTSeq-count results for RNA-seq data. Download Table S1, XLSX file, 0.01 MB. Copyright © 2022 Kim et al. 2022 Kim et al. https://creativecommons.org/licenses/by/4.0/ This content is distributed under the terms of the Creative Commons Attribution 4.0 International license.

### Estimation of ancestral gene expression of single-copy orthologs.

To investigate transcriptional divergence of homologous genes conserved in the five sordariomycete fungi during perithecial development, we identified 3,942 single-copy orthologous gene clusters (SCOGs) with representation in all five species by clustering orthologous gene sets. Of 3,942 SCOGs, we excluded 78 SCOGs that include one or more genes with average values for *Z*-transformed reads per kilobase of transcript per million mapped reads (RPKM) lower than −3 throughout the six developmental stages (S0 to S5), which are considered not actively transcribed (47) (Table 1) (see Materials and Methods). Then, we estimated ancestral gene expression levels for the expressed SCOGs to identify genes whose expression increased substantially in one lineage during the divergence of the five species from the MRCA. Since absolute expression levels such as RPKM values generated by RNA-seq cannot be used for interspecies comparisons (10, 48), we used fold change of expression levels between each adjacent pair of developmental stages as a continuous variable for estimating gene expression levels for the MRCA (10). We calculated fold change differentials (FCD), subtracting the estimated fold change values of the MRCA from the observed fold change values of extant species for every SCOG. By doing so, we generated lists of prioritized genes for targeted knockout experiments in F. graminearum, which may have undergone transcriptional activation during the evolutionary processes in one or more developmental stages (Table 2; see Data Set S1 for the other four species).

**TABLE 1:** Summary of transcriptome data during perithecial development

Parameter^*a*^	Result for^*b*^:				
	*F. graminareum*	*F. neocosmosporiellum*	M. oryzae	N. crassa	*C. globosum*
No. of annotated genes	14,164	14,353	12,593	9,758	11,124
No. of genes expressed (zRPKM value of >−3)	12,669	12,672	11,048	9,233	10,409
% of genes expressed	89.4	88.3	87.7	94.6	93.6

a The transcriptome data include 3,942 single-copy orthologs (SCOGs), and 3,864 (98%) of the SCOGs were expressed.

b Shown are results for Fusarium graminearum, Fusarium neocosmosporiellum, Magnaporthe oryzae, Neurospora crassa, and Chaetomium globosum.

**TABLE 2:** Top-ranked genes with the largest evolved increases in gene expression and their knockout phenotypes

Gene ID^*a*^	FCD (stages)^*b*^	Putative function (gene product)	Knockout phenotype	Rank	Source or reference
FGRRES_17170	80.3 (S0−S1)	Oxidoreductase (PGLX)	Perithecia pigment	1	Frandsen et al. (51)
FGRRES_02305	42.2 (S0−S1)	Aminohydrolase	Wild type	3	This study
FGRRES_00262	13.4 (S0−S1)	Hypothetical protein	Wild type	14	This study
FGRRES_08061	10.2 (S0−S1)	Phosphoenolpyruvate carboxykinase	Wild type	18	This study
FGRRES_00348	9.5 (S0−S1)	Argonaute 2 (AGO2)	Fewer asci/ascospore	22	Kim et al. (24)
FGRRES_08576	9.5 (S0−S1)	Meiotic cohesin (yeast rec8)	Aborted and less ascospores	23	This study
FGRRES_00503	18.7 (S1−S2)	*O*-Succinylhomoserine lyase	Wild type	11	This study
FGRRES_03365	13.9 (S1−S2)	Nuclease/phosphatase family	Wild type	13	This study
FGRRES_10126	11.5 (S1−S2)	Propionyl-CoA synthetase	Wild type	16	This study
FGRRES_05439	10.1 (S1−S2)	Hypothetical protein	Wild type	19	This study
FGRRES_06533	9.6 (S1−S2)	Hypothetical protein	No perithecia	20	This study
FGRRES_06797	9.6 (S1−S2)	Hypothetical protein	No paraphyses and asci	21	This study
FGRRES_03356	9.5 (S1−S2)	Hypothetical protein	Wild type	24	This study
FGRRES_06285	7.5 (S1−S2)	α/β-Hydrolase superfamily	No paraphyses and asci	36	This study
FGRRES_16442	41.4 (S2−S3)	Oxidoreductase	Wild type	4	This study
FGRRES_09307	31.1 (S2−S3)	Hypothetical protein	Aborted and less ascospores	7	This study
FGRRES_07376	22.0 (S2−S3)	Copper homeostasis protein	Wild type	9	Trail et al. (10)
FGRRES_09973	20.6 (S2−S3)	Tyrosine phosphatase	Wild type	10	This study
FGRRES_00526	11.1 (S2−S3)	Dihydroneopterin aldolase	Wild type	17	This study
FGRRES_01058	56.4 (S3−S4)	Serine threonine kinase (PUK1)	Abnormal ascospores	2	Wang et al. (21)
FGRRES_17271	35.8 (S3−S4)	RNA-binding protein (yeast spo5)	No ascospore	6	This study
FGRRES_04345	29.5 (S3−S4)	Aspartic protease	Wild type	8	This study
FGRRES_05400	18.4 (S3−S4)	Glycoside hydrolase family 16	Defects in ascospore release	12	This study
FGRRES_10224	13.2 (S3−S4)	Hypothetical protein	Wild type	15	This study
FGRRES_17505	9.1 (S3−S4)	Asparaginase family protein	Defects in ascospore release	25	This study
FGRRES_17508	7.7 (S3−S4)	Hypothetical protein	No ascospore	35	This study
FGRRES_17498	39.9 (S4−S5)	sugar transporter	Wild type	5	This study

a Gene ID based on the Ensembl annotation version 32 of Fusarium graminearum wild-type strain PH-1.

b Fold change differentials (FCD) were calculated as max [(*X_t+1_* – *X_t_*) − (*Y_t+1_* – *Y_t_*)], where *t* represents the developmental stages (0 to 4), *X* is the LOX-transformed expression level of an extant species (e.g., F. graminearum), and *Y* is the inferred expression level of the most recent common ancestor (MRCA) of the five species: *C. globosum*, F. graminearum, *F. neocosmosporiellum*, M. oryzae, and N. crassa.

10.1128/mbio.01100-22.8 DATA SET S1 Top 25 genes with the largest evolved increases in gene expression in M. oryzae, N. crassa, *F. neocosmosporiellum*, and *C. globosum*. Download Data Set S1, XLSX file, 0.1 MB. Copyright © 2022 Kim et al. 2022 Kim et al. https://creativecommons.org/licenses/by/4.0/ This content is distributed under the terms of the Creative Commons Attribution 4.0 International license.

### Functionally conserved genes that have undergone transcriptional divergence.

**FIG 2:**
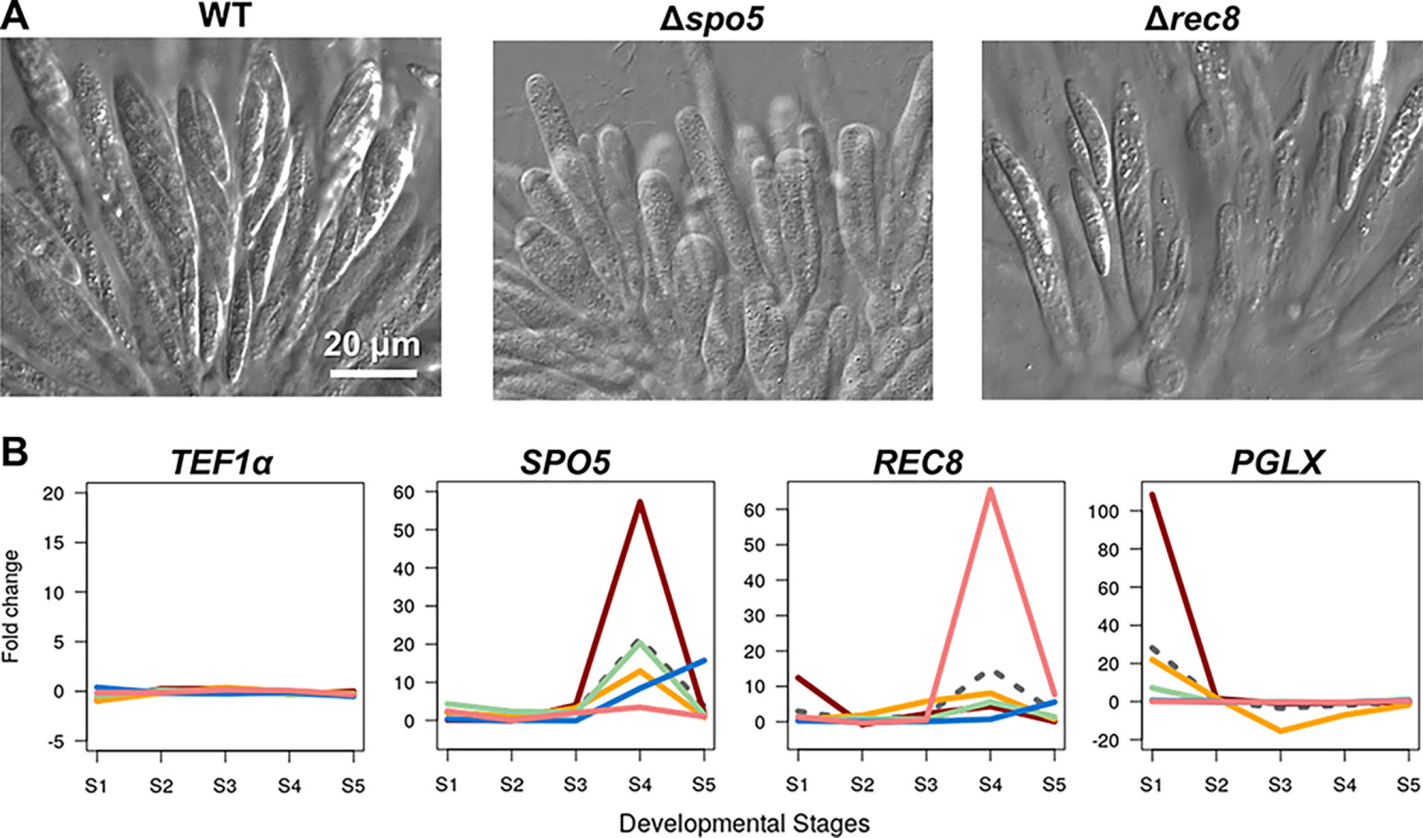
Phenotypic analysis of conserved functional genes. (A) Ascus and ascospore morphology of the wild-type strain (WT) and Fusarium graminearum *SPO5* and *REC8* knockout mutants. (B) Fold changes of expression levels during perithecial development. Note that the expression level of *TEF1α* (translation elongation factor 1-α) remains constant throughout the developmental stages (S0 to S5) in the five species. Red line, F. graminearum; orange line, *F. neocosmosporiellum*; blue line, Neurospora crassa; green line, Magnaporthe oryzae; pink line, Chaetomium globosum; dashed line, fold changes of inferred expression levels in the most recent common ancestor for the five species.

In F. graminearum, knockouts were completed on the 25 top-ranked genes that exhibited FCD ranging from 9 to 80 (Table 2). These top-ranked genes were predicted to have evolved increases in gene expression at one or more developmental stages. Of these, we conducted functional characterization for 21 genes that have not been previously studied and found that knockout mutants for 10 genes showed defective phenotypes during perithecial development, while knockout mutants for 15 genes produced normal perithecia and ascospores (Table 2; Fig. S1). The *PUK1* (perithecium unique kinase 1) and *AGO2* (argonaute 2) genes in F. graminearum were reported to exhibit knockout phenotypes defective in ascospore formation (21, 24). In addition, there were orthologs of *rec8* (yeast meiotic recombination protein 8) and *spo5* (yeast sporulation-specific protein 5) in the prioritized list (Table 2). The meiotic cohesin gene *rec8* and RNA-binding protein gene *spo5* play crucial roles during the first and second meiotic divisions, respectively, in fission yeast (49, 50). Similar to the knockout phenotypes observed in fission yeast, the Δ*spo5* strain was devoid of ascospores in F. graminearum (Fig. 2A), presumably due to failure in ascospore wall formation as with the yeast knockout (49). The F. graminearum Δ*rec8* strain produced fewer than eight ascospores per ascus (Fig. 2A), consistent with the role of the ortholog in accurate chromosome segregation during meiosis II in yeast (50). Despite the conserved roles of *rec8* and *spo5*, there was transcriptional divergence among the five species. The *spo5* orthologs in the five species were induced at S4 when meiosis takes place, but the degree of induction for the *spo5* ortholog in F. graminearum was most prominent (Fig. 2B). For *rec8*, it was induced at S1 when the sexual stage had just begun in F. graminearum, and the ortholog in *C. globosum* exhibited the greatest induction at S4 (Fig. 2B).

10.1128/mbio.01100-22.1 FIG S1 Perithecial morphology of knockout mutants for the 25 top-ranked genes. Download FIG S1, PDF file, 0.3 MB. Copyright © 2022 Kim et al. 2022 Kim et al. https://creativecommons.org/licenses/by/4.0/ This content is distributed under the terms of the Creative Commons Attribution 4.0 International license.

### Transcriptional divergence of perithecial pigment genes.

Perithecial development in sordariomycete fungi is often tightly associated with the production of pigments. *PGLX*, a member of the bostrycoidin biosynthesis gene cluster responsible for the black perithecial pigmentation in F. graminearum (51, 52), exhibited the greatest FCD in the prioritized gene list (Table 2). The role of the *PGLX* in the bostrycoidin biosynthesis is not clear in F. graminearum (51). However, a knockout mutant lacking the *PGLX* ortholog in a related species, Fusarium fujikuroi, produces more fusarubin (a bostrycoidin analogue) as a perithecial pigment, suggesting that *PGLX* is involved in the biosynthesis of the perithecial pigments in Fusarium species (53). *PGL1*, *PGLJ*, and *PGLM* are necessary and sufficient for the biosynthesis of bostrycoidin in F. graminearum (51). Although the three core biosynthetic genes were conserved and syntenic between F. graminearum and *F. neocosmosporiellum* (68 to 81% identity at protein sequence levels), the *PGLX* ortholog in *F. neocosmosporiellum* was located distal to the three core biosynthetic genes, interspersed with 17 genes, as in a related species, F. solani (51, 53). Although the *PGLX* orthologs were found in the genomes of *C. globosum*, M. oryzae, and N. crassa, the three core biosynthetic genes were absent in these three species, which are known to produce a black perithecial pigment of melanin origin (45, 54).

**FIG 3:**
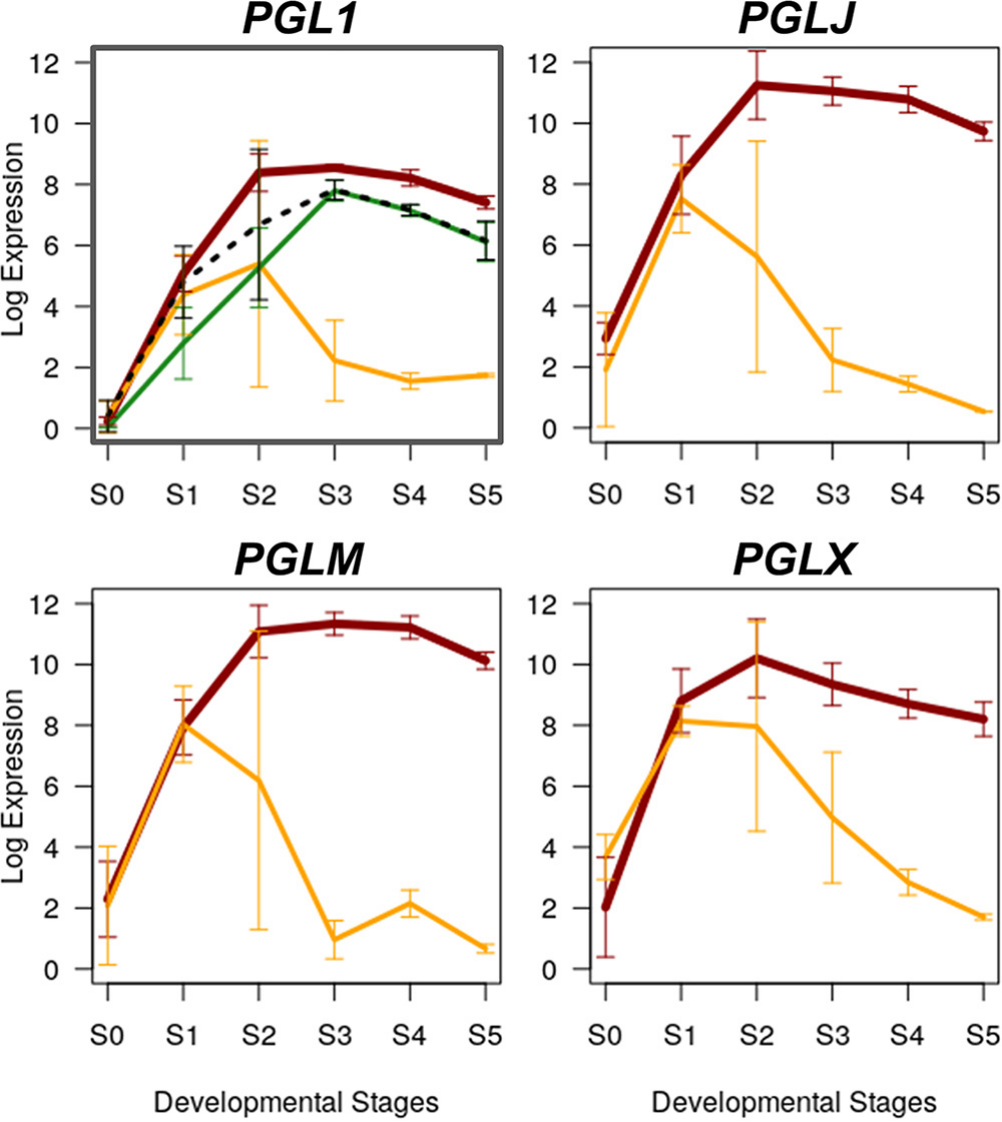
Transcriptional divergence of perithecial pigment genes. Expression levels of the three core biosynthetic genes (*PGL1*, *PGLJ*, and *PGLM*) and *PGLX*, involved in production of bostrycoidin, the black pigment deposited in perithecial walls in Fusarium graminearum. Expression levels are presented as log_2_ (RPKM + 1): red line, F. graminearum; orange line, *F. neocosmosporiellum*. The green line in the *PGL1* panel depicts expression levels of the *PKSN* involved in the biosynthesis of red perithecial pigment in *F. neocosmosporiellum*, and the dashed line depicts a combined expression level of the *PGL1* and *PKSN* genes in *F. neocosmosporiellum*. Error bars indicate standard deviation of mean of three replicates.

*PGLX* expression was highly induced at S1 in both F. graminearum and *F. neocosmosporiellum*, whereas the expression levels of the *PGLX* orthologs were largely unchanged throughout the developmental stages in *C. globosum*, M. oryzae, and N. crassa (Fig. 2B). The *PGLX* expression level gradually declined after S1 in *F. neocosmosporiellum*, while *PGLX* remained highly expressed in F. graminearum (Fig. 3). The three core biosynthetic genes exhibited similar expression patterns, with the *PGLX* expression in F. graminearum and *F. neocosmosporiellum* indicating a coordinated regulation of the core biosynthetic gene cluster with *PGLX* (Fig. 3). Previously, we identified the *PKSN* gene (also known as *PKS35*) as coding for a polyketide synthase (PKS) responsible for the biosynthesis of red perithecial pigment in *F. neocosmosporiellum* (44). The *PKSN* gene was induced at S1 and remained highly expressed after S2 in *F. neocosmosporiellum*, consistent with its role in perithecial wall pigmentation, while the expression level of the *PGL1* ortholog steeply declined after S2, suggesting a divergent role of the bostrycoidin/fusarubin pigment system in *F. neocosmosporiellum* (Fig. 3).

### Identification of novel genes important for perithecial development in F. graminearum.

**FIG 4:**
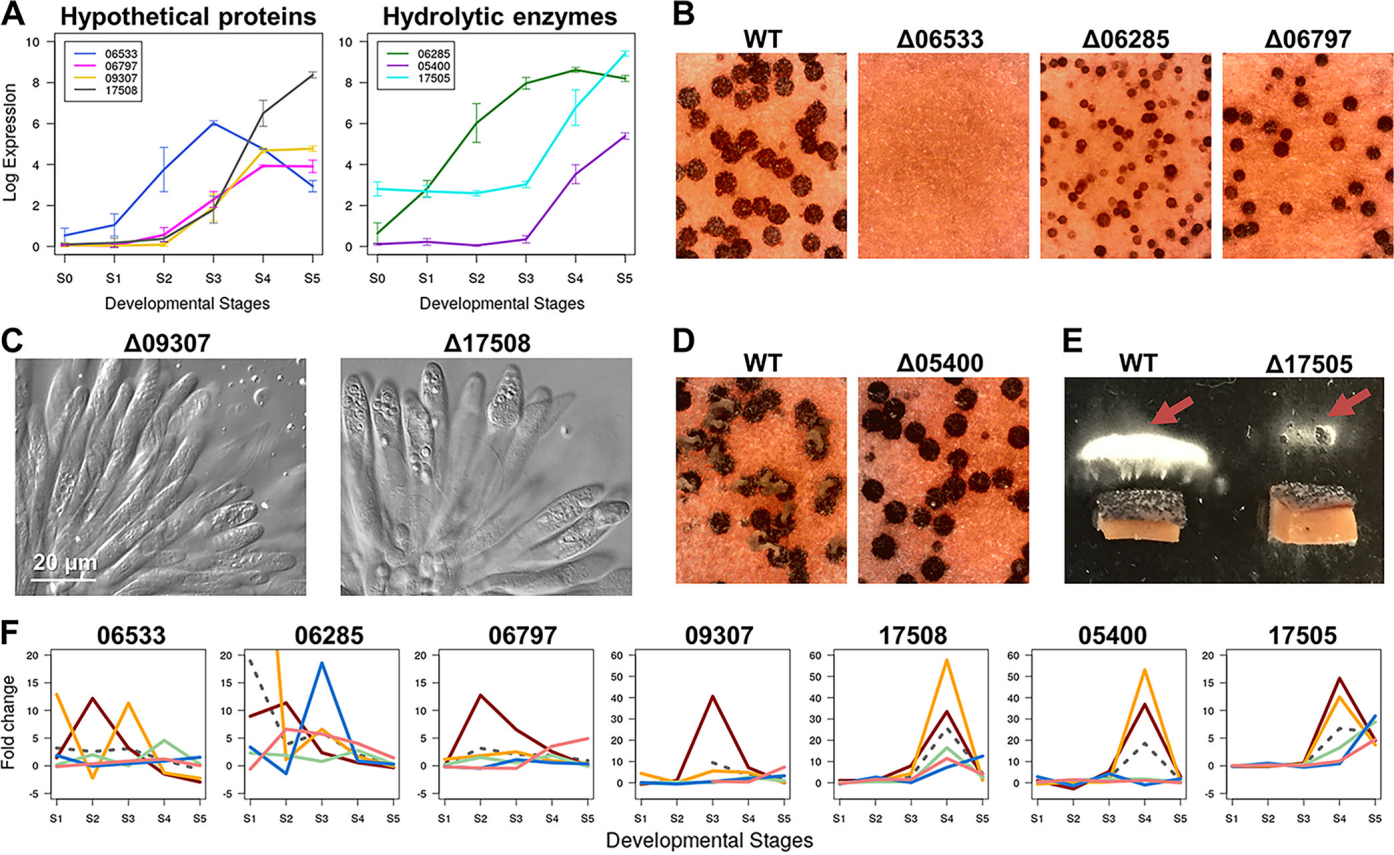
Novel genes encoding hypothetical proteins involved in different stages of perithecial development. (A) Expression levels of genes encoding novel hypothetical proteins and hydrolytic enzymes with unknown function during perithecial development. Expression levels are presented as log_2_ (RPKM + 1). Error bars indicate the standard deviation of the mean of three replicates. (B) Knockout phenotypes of novel genes (FGRRES_06285, -_06533, and -_06797) encoding hypothetical proteins. Perithecia (black spheres) formed on carrot agar. Photos were taken 7 days after sexual induction. (C) Ascus morphology in FGRRES_09307 and -_17508 knockout mutants. Note the barren asci of the mutants, lacking ascospores. For the ascus morphology of the wild-type strain, refer to Fig. 2A. (D) The wild-type strain exudes cirrhi (ascospores exuded *en mass*e from the ostioles of perithecia). No cirrhi were produced in FGRRES_05400 knockout mutants. Photos were taken 14 days after sexual induction. (E) Reduced ascospore discharge in the FGRRES_17505 knockout mutant. Agar plugs supporting mature perithecia (across the bottom of each panel) were oriented perpendicular to the glass slides so that spores fired and accumulated on the slides (red arrows). Photos taken 3 days after the set up. (F) Fold changes of expression levels of genes encoding hypothetical proteins and hydrolytic enzymes during perithecial development. Red line, F. graminearum; orange line, *F. neocosmosporiellum*; blue line, Neurospora crassa; green line, Magnaporthe oryzae; pink line, Chaetomium globosum; dashed line, fold changes of inferred expression levels in the most recent common ancestor for the five species.

By phenotyping knockouts of the ranked genes in F. graminearum, we identified seven genes that play crucial roles at different stages of perithecial development: four genes (FGRRES_06533, -_06797, -_09307, and -_17508) are not functionally annotated and do not encode any predicted protein domains (henceforth referred as “hypothetical genes”), and three genes (FGRRES_05400, -_06285, and -_17505) encode different hydrolytic enzymes of unknown function (Table 2). All of these genes were highly induced during perithecial development (Fig. 4A). Knockouts of three genes (FGRRES_06285, -_06533, and -_06797) were arrested at earlier developmental stages before the development of asci. These knockouts of genes functioning in early developmental stages included one that was devoid of perithecia (FGRRES_06533) and two that were arrested at protoperithecium formation (FGRRES_06285 and _06797) (Fig. 4B; Fig. S2). Knockouts of the other four genes exhibited defective phenotypes at developmental stages after the development of asci. These knockouts of genes functioning in later developmental stages included two that produced no ascospores in fully developed asci (FGRRES_09307 and -_17508) (Fig. 4C) and two that produced normal ascospores, but exhibited no formation of cirrhi and reduced spore firing (FGRRES_05400 and -_17505) (Fig. 4D and E; Fig. S2).

10.1128/mbio.01100-22.2 FIG S2 Sexual phenotypes of novel genes encoding hypothetical proteins and hydrolytic enzymes. Download FIG S2, PDF file, 0.2 MB. Copyright © 2022 Kim et al. 2022 Kim et al. https://creativecommons.org/licenses/by/4.0/ This content is distributed under the terms of the Creative Commons Attribution 4.0 International license.

The observed knockout phenotypes of hypothetical genes largely coincided with the evolved increase in gene expression in F. graminearum (Fig. 4F). Notably, the two hypothetical genes (FGRRES_06797 and -_09307) have evolved to be highly induced at S2 and S3, respectively, in F. graminearum. The expression of a hypothetical gene (FGRRES_17508) and two genes encoding hydrolytic enzymes (FGRRES_05400 and -_17505) was highly induced at S4 in both F. graminearum and *F. neocosmosporiellum* (Fig. 4F), suggesting coexisting roles of these genes in ascospore production and firing in the Fusarium lineage. For the two genes (FGRRES_06285 and -_06533) whose knockouts affected earlier developmental stages in F. graminearum, there was no shared pattern of expression among the homologous genes. All of the above-described knockouts exhibited normal hyphal and conidial morphologies during vegetative growth, suggesting sexual-stage-specific functions of these novel genes.

### Transcriptional divergence of hypothetical proteins.

**FIG 5:**
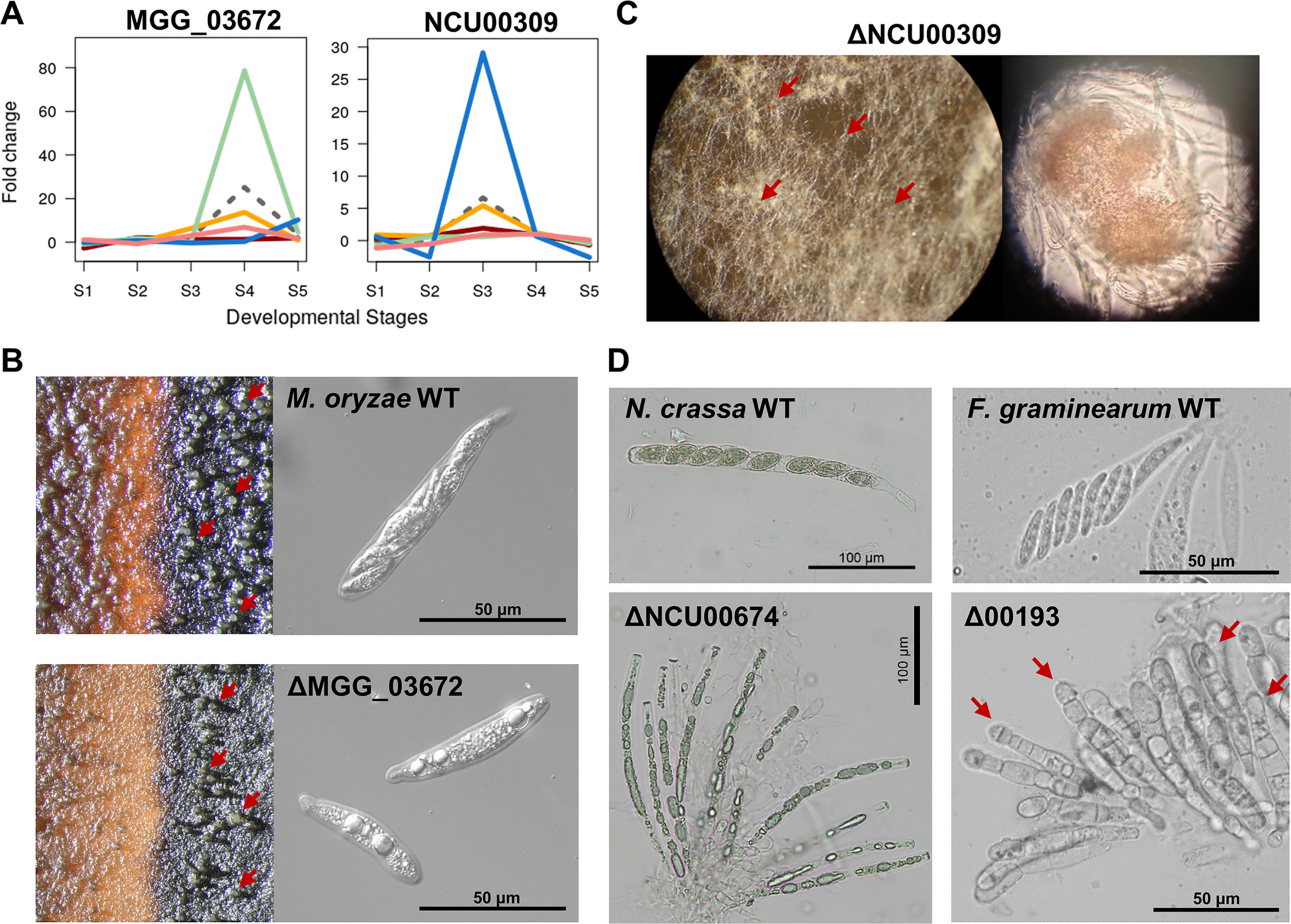
Functionally diverged genes encoding hypothetical proteins in Magnaporthe oryzae and in Neurospora crassa. (A) Fold changes of expression levels of novel genes encoding hypothetical proteins during perithecial development. Red line, F. graminearum; orange line, *F. neocosmosporiellum*; blue line, Neurospora crassa; green line, Magnaporthe oryzae; pink line, Chaetomium globosum; dashed line, fold changes of inferred expression levels in the most recent common ancestor for the five species. (B) Beaks of perithecia protruding from the melanized stroma (red arrows in left panels) and ascus morphology (right panels) of the wild-type strain (WT) and knockout mutants of a hypothetical gene (MGG_03672) in M. oryzae. (C) Knockout phenotypes of a hypothetical gene (NCU00309) in N. crassa. Perithecial formation halted at a stage of protoperithecial development (red arrows in left panel) and an enlarged view of a squashed protoperithecium devoid of cellular contents (right panel). (D) Knockout phenotypes of the MGG_03672 orthologs in N. crassa (NCU00674) and in F. graminearum (FGRRES_00193). Red arrows indicate abnormal asci with irregular septations, lacking ascospores.

Since we discovered novel developmental roles of numerous hypothetical genes that have been transcriptionally activated during perithecial development in F. graminearum, we investigated hypothetical genes found in other evolutionary lineages in the Sordariomycetes, such as M. oryzae and N. crassa. Among the 25 top-ranked genes, there were 10 hypothetical genes in M. oryzae and 7 in N. crassa (Data Set S1). We generated knockout mutants for all the 10 hypothetical genes in M. oryzae and obtained knockout mutants for six hypothetical genes in N. crassa, available from the genome-wide knockout project of N. crassa (19, 55). Since M. oryzae and N. crassa require outcrossing to form fully developed perithecia, we crossed two knockout mutants with opposite mating types on carrot agar medium to observe perithecial development in M. oryzae and N. crassa. Two hypothetical genes, MGG_03672 in M. oryzae and NCU00309 in N. crassa, were estimated to have evolved increases in gene expression at S4 and S3, respectively (Fig. 5A). The knockout phenotypes for MGG_03672 and NCU00309 coincided with their elevated expression at S4 and S3, respectively: crossing the knockouts of MGG_03672 produced perithecia with immature asci containing a few ascospores with abnormal shape (Fig. 5B), and crossing the knockouts of NCU00309 produced protoperithecia that failed to develop further (Fig. 5C).

To examine changes in developmental roles of hypothetical genes MGG_03672 and NCU00309 in M. oryzae and N. crassa, we compared knockout phenotypes of their orthologous genes in F. graminearum. Similar to the knockout phenotype of MGG_03672, knockouts of the N. crassa ortholog of MGG_03672 (NCU00674) produced abnormal ascospores (Fig. 5D). On the other hand, knockouts of the F. graminearum ortholog of MGG_03672 (FGRRES_00193) produced asci with irregular septations, which are devoid of ascospores (Fig. 5D). For the NCU00309 gene, whose knockouts were arrested at the protoperithecial stage, knockouts of the F. graminearum ortholog (FGRRES_06775) produced normal asci and ascospores (Fig. S3).

10.1128/mbio.01100-22.3 FIG S3 Sexual phenotypes of FGRRES_06775 homologous to NCU00309. Download FIG S3, PDF file, 0.2 MB. Copyright © 2022 Kim et al. 2022 Kim et al. https://creativecommons.org/licenses/by/4.0/ This content is distributed under the terms of the Creative Commons Attribution 4.0 International license.

### Transcriptional divergence of hypothetical genes.

**FIG 6:**
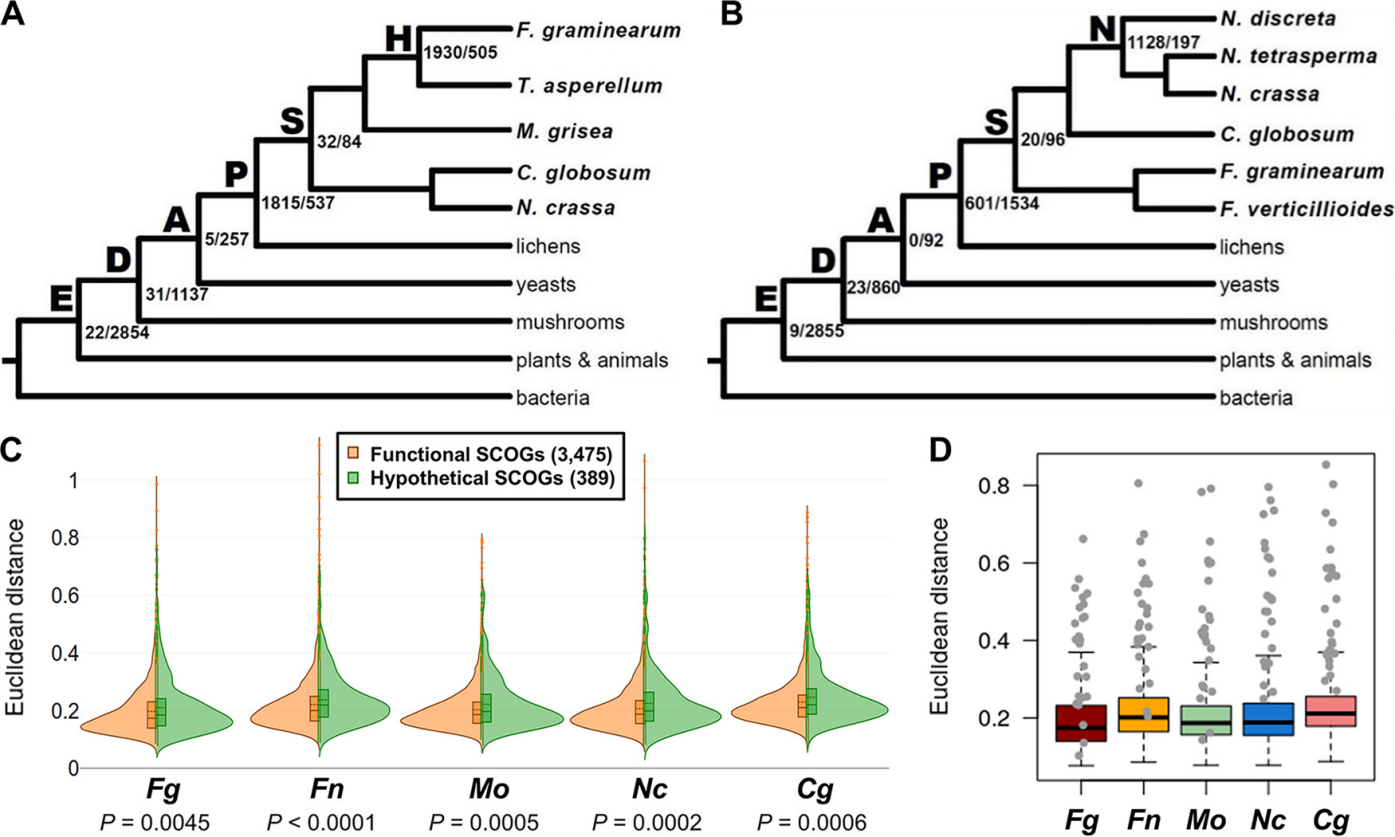
Transcriptional divergence of genes encoding hypothetical proteins. (A and B) Phylostratigraphic gene ages of the annotated genes in the F. graminearum and N. crassa genomes. The numbers of annotated genes (“hypothetical gene/functional gene”) are indicated next to nodes of the phylogenies. E, Eukaryotes; D, Dikarya; A, Ascomycota, P, Pezizomycotina; S, Sordariomycetes; H, Hypocreaceae; N, *Neurospora*. (C) The degrees of expression divergence for single-copy ortholog clusters (SCOGs) were estimated by averaged Euclidean distance between every possible pairwise comparison of relative expression levels during perithecial development in the five species. Shown are violin plots of Euclidean distance for 3,864 SCOGs that were assigned as “functional SCOGs” if at least two genes in each SCOG are annotated by the InterproScan program (E value, <10^−10^) but otherwise assigned as “hypothetical SCOGs.” Horizontal lines and dashed lines in the box plots indicate average and median values, respectively. Fg, Fusarium graminearum; Fn, *F. neocosmosporiellum*; Mo, Magnaporthe oryzae; Nc, Neurospora crassa; Cg, Chaetomium globosum. *P* values for Mann-Whitney *U* test statistics were shown below the species name. (D) Box plots of Euclidean distance for 3,864 SCOGs. The 25 top-ranked genes in the priority gene lists are depicted by gray dots.

It was shown that deeply conserved genes (“old genes”), as well as newly emerged genes (“young genes”) tend to be expressed during specific developmental stages in fungi, playing important roles in sculpting fruiting body morphologies (56). To assess the ages of the annotated genes in the F. graminearum and N. crassa genomes, we sorted hypothetical genes and genes with predicted functional domains (henceforth referred as “functional genes”) using a phylostratigraphic approach, in which ages of the phylogenetically classified genes were assigned based on the set of species that share orthologous genes. For this, we generated two different phylogenetic trees with different sets of terminal clades (Fig. 6A and B). The hypothetical genes exhibited similar distribution patterns between the two genomes, with the largest numbers of hypothetical genes observed in the most recent divergences: 1,930 hypothetical genes (21% of a total of 9,209 genes) shared between F. graminearum and Trichoderma asperellum, which belong to the order Hypocreales (Fig. 6A), and 1,128 hypothetical genes (15% of total of 7,415 genes) shared between three *Neurospora* species (Fig. 6B). The second-largest numbers of hypothetical genes were observed with the emergence of the subphylum Pezizomycotina (Fig. 6A and B; indicated by “P”), which encompasses many fungal lineages with diverse fruiting body morphologies. On the other hand, a majority of functional genes were classified as “old genes” and were conserved among Eukaryotes and Dikarya, accounting for 43% and 50% of the total genes in the F. graminearum and N. crassa genomes, respectively.

In this study, we identified many hypothetical genes whose knockouts exhibited arrested development at a stage when their expression has substantially increased, compared to their inferred MRCA with their closest relative in this study. To compare degrees of transcriptional divergence of functional genes and hypothetical genes between different fungi, we classified 3,864 SCOGs of the five species into 3,475 SCOGs annotated by the InterProScan program (E value, <10^−10^ [referred as functional SCOGs]) and 389 SCOGs lacking any known functional protein domains (E value, ≥10^−10^ [referred as hypothetical SCOGs]) (see Materials and Methods). Euclidean distance of relative expression levels across the six developmental stages (S0 to S5) was calculated between orthologs and averaged for every SCOG as a measure of degree of expression divergence. Overall, hypothetical SCOGs tend to be more diverged in gene expression than functional SCOGs in all the five species (Mann-Whitney *U* test, *P* < 0.01) (Fig. 6C). Also, we found that the expression divergence of the 25 top-ranked genes in the five species was generally high (Fig. 6D), indicating that our method is highly efficient for identifying genes that have undergone transcriptional divergence during adaptive radiation of fungal lineages.

## DISCUSSION

In this study, we identified genes likely to play a diverging role during conserved development in sexual reproduction among fungi in the Sordariomycetes, based on evolved changes in expression of conserved orthologous genes. Taking advantage of highly annotated fungal genomes and efficient gene knockout methods in three model systems—F. graminearum, M. oryzae, and N. crassa—for studying sexual development and pathogenomics, we systematically analyzed knockout phenotypes of genes that are phylogenetically inferred to have experienced the largest increases in gene expression after divergence from the MRCA. We initially aimed to identify genes responsible for the evolution of the complex phenotypes observed in these three species. However, in this study, we did not identify such genes. The complex phenotypes may be controlled by coordinated expression of multiple genes, which cannot be identifiable by single-gene knockout studies. Nevertheless, we found that the observed phenotypic defects of knockout mutants of hypothetical genes were largely coincident with transcriptional shifts of the genes, which are crucial for achieving successful sexual development in each lineage. Functional analyses of these evolved genes further illustrated the utility of this evolutionary approach in the identification of hitherto uncharacterized genes important for certain developmental processes. Overall, hypothetical genes are relatively younger than functional genes whose orthologs have been characterized and extensively studied in other eukaryotic model organisms. Hypothetical genes are often fungal or lineage specific, and thus have often been less well characterized than deeply conserved genes at higher taxonomic levels. Although greater degrees of transcriptional divergence of hypothetical genes may be attributable to neutrally arising expression variation widely observed in young genes because of weaker evolutionary constraint (56), our systemic knockout studies for hypothetical genes demonstrated that these hypothetical genes have evolved to be necessary for successful sexual development, showing significant shifts in their expression levels in certain groups of fungi.

### Transcriptionally diverged genes with hypothetical function.

Large-scale knockout projects have focused mainly on specific functional groups of genes, such as kinases, phosphatases, and transcription factors. These gene families are involved in cellular signaling and regulation of gene expression, many of which have dramatic impacts on perithecial development (21–23). In the five species investigated in this study, 24 to 29% of expressed genes in our transcriptome data sets were predicted to have no known protein domains, using the InterProScan program, which makes it challenging to efficiently guide the knockout phenotyping without predicted functions of those gene products. Our functional analyses for the top 25 genes in F. graminearum, M. oryzae, and N. crassa revealed that the peak expression of the hypothetical genes during perithecial development is frequently coincident with the developmental stage of appearance of the defective knockout phenotypes. Notably, expression of one hypothetical gene (FGRRES_06533) increased markedly at early developmental stages, and the knockouts aborted the initiation of perithecial development in F. graminearum. However, knockouts of the orthologous gene in N. crassa (NCU08555) exhibited the wild-type morphology with perithecia developing to full maturity. The deduced amino acid sequence contained a partial transmembrane domain of the LrgB superfamily (E value, ≥10^−10^) that was shown to be involved in cell death and lysis in bacteria (57) and in chloroplast development in Arabidopsis thaliana (58). Heterologous expression of a plant LrgB ortholog in budding yeast increased the membrane permeability of yeast cells (58), suggesting a possible role of FGRRES_06533 in controlling membrane integrity during the early developmental stage in F. graminearum. In a second example, expression of one hypothetical gene (FGRRES_06796) was greatly shifted, peaking at S2, when perithecial wall formation initiates in F. graminearum. Concordantly, the knockout phenotype was arrested at the protoperithecial stage. The orthologous gene in N. crassa (NCU06680) did not show a similar shift in expression and the knockout produced normal perithecia.

We identified two hypothetical genes (FGRRES_09307 and -_17508) whose knockouts produced apparently normal asci containing no ascospores. However, knockouts of the N. crassa orthologs (NCU04067 and NCU03562) exhibited wild-type perithecial phenotypes, suggesting that the F. graminearum orthologs have adopted nonredundant, essential roles during meiosis, whereas the N. crassa orthologs either no longer have roles or now have nonessential or redundant roles. It is noteworthy that N. crassa is among a few model fungi that have lost key genes for the standard meiosis toolkit equipped in most organisms—in this case *DMC1* and *RAD51* (59, 60). The hypothetical gene FGRRES_09307 was annotated as coding for a nuclear fusion protein in FgMutantDB (61), which is similar to the yeast gene *kar5*, which is involved in karyogamy (62). Although exhibiting only 19% protein sequence identity with the yeast *kar5*, FGRRES_09307 contains two predicted transmembrane domains at the C-terminus region, as in the yeast *kar5* gene (62). Given the aborted ascospore production in the knockouts, FGRRES_09307 may have retained its ancestral function related to karyogamy. In addition, we identified two genes that affected ascospore release: FGRRES_05400 encodes a protein that belongs to glycoside hydrolase family 16 (GH16), and FGRRES_17505 encodes an asparaginase. Little is known about the developmental roles of GH16 and asparaginase in filamentous fungi, but some GH16 enzymes are important for proper formation of yeast cell walls or ascospore walls in budding yeast (63, 64). Expression of FGRRES_05400 and FGRRES_17505 was specifically induced at S4, concomitant with production of asci and ascospores, suggesting that their roles are relegated to sexual development.

Of the 10 hypothetical genes in the prioritized list for M. oryzae, we identified a hypothetical gene (MGG_03672) that is crucial for ascospore production. The observed knockout phenotypes for the orthologous genes were similar between M. oryzae, and N. crassa. However, the knockout phenotype of the orthologous gene in F. graminearum (FGRRES_00193) was different from those observed in M. oryzae, and N. crassa, phylogenetically distantly related species, suggesting that the role of the hypothetical gene in ascospore development has slightly diverged in sordariomycete fungi. Of the six hypothetical genes in the prioritized list for N. crassa, knockouts of one hypothetical gene, NCU00309, failed to develop perithecial walls, being arrested in the protoperithecial stage. The deduced amino acid sequence of NCU00309 contained a wall stress-responsive component (WSC) domain. Fungal proteins containing WSC domains play a role in agglutination of fungal cells (65) and maintaining cell wall integrity (66), suggesting the role of NCU00309 in development of perithecial walls. Despite its essential role in N. crassa, the phenotype of an F. graminearum strain with FGRRES_06775 knocked out was normal, suggesting that WSC-containing genes in N. crassa may have a reinforced role enabling the development of thicker and sturdier perithecial walls than other fungal species do.

### Evolutionary perspectives on perithecial pigment production.

Fungi belonging to the Nectriaceae produce pigmented perithecia: dark purple to black perithecia in *Gibberella* teleomorphs (associated with F. graminearum) and orange to red perithecia in *Nectria*–like teleomorphs (associated with *F. neocosmosporiellum*) (67, 68). Frendesen et al. (51) proposed evolutionary models for pigment production of species in the Nectriaceae, in which they advanced a hypothesis that bostrycoidin/fusarubin is likely the ancestral pigment system of Fusarium and allied genera, in relation to red perithecial pigment produced by species connected to *Nectria*–like teleomorphs and other mycelial pigments, such as aurofusarin and bikaverin. The red perithecial pigment in *F. neocosmosporiellum* appears to be a derived character, as homologous *PKSN* gene clusters are found exclusively in species of *Nectria*-like teleomorphs, while *PGL1* gene clusters for the biosynthesis of bostrycoidin/fusarubin are ubiquitous in fusaria (69). Bostrycoidin/fusarubin and its analogues may have been originally utilized as both mycelium and perithecium pigments, as reflected by the several observations about their roles in development. (i) Fusarubin, the perithecial pigment of *F. fujikuroi* (teleomorph Gibberella fujikuroi), accumulates in mycelia under conditions where the mycelial pigment bikaverin is not produced (53). (ii) Similarly, knockouts of *AUR1* responsible for production of aurofusarin, the major mycelial pigment in F. graminearum (teleomorph Gibberella zeae), cause accumulation of black pigments in the stroma, which are likely derived from bostrycoidin (52). (iii) Finally, fusarubin is one of the major mycelial pigments in many species connected to *Nectria*-like teleomorphs, including F. solani (70).

In this study, we observed transcriptional divergence of the *PGL1* gene clusters (including *PGLX*) between F. graminearum (*Gibberella* teleomorph) and *F. neocosmosporiellum* (*Nectria*-like teleomorph): expression of the *PGL1* gene cluster remains highly upregulated across perithecial developmental stages in F. graminearum; however, the *PGL1* ortholog in *F. neocosmosporiellum* is downregulated after protoperithecia become visible. It is possible that the acquisition of another pigment system (i.e., the *PKSN* gene cluster) for the perithecial wall pigment in *F. neocosmosporiellum* led to shifts in the expression levels of the *PGL1* gene clusters during perithecial development to properly accommodate the two distinct anabolic pigmentation pathways. A recent study showed that a transcription factor affects expression of these two distinct pigment systems in F. solani (*Nectria-*like teleomorph), suggesting the regulation of *PGL1* and that of the *PKSN* gene clusters are somehow connected (71). Interestingly, the sum of the *PGL1* and *PKSN* expression levels in *F. neocosmosporiellum* approximates the *PGL1* expression level in F. graminearum across the developmental stages (Fig. 3C). This situation resembles quantitative subfunctionalization, referring to the phenomenon where aggregate expression levels of paralogous genes with redundant, but more specialized, functionality approximates the expression level of their preduplicated gene in other species (72). This phenomenon supports the “division of labor” hypothesis for mycelial and perithecial pigmentation in species connected to *Nectria*-like teleomorph, including *F. neocosmosporiellum* (51). Unlike Fusarium and allied genera, a dihydroxynaphthalene-derived melanin is the perithecial pigment in *C. globosum* and N. crassa, as well as in other sordariomycete fungi (73, 74). A polyketide synthase (PKS) gene, *per-1*, is responsible for melanization of both perithecial wall tissues and ascospores in N. crassa (54, 75). Interestingly, the *per-1* ortholog (dubbed *ALB1*) in M. oryzae is involved in melanization of appressoria, specialized infection structures for plant infection (76, 77), indicating that fungal pigments are produced in tissue-specific manners and have evolved to have divergent roles. The PKS genes *per-1*, *PKSN*, and *PGL1* belong to nonreducing type PKS groups II, III, and IV, respectively (78), indicating that different lineages of sordariomycete fungi have adopted distinct PKS types for perithecial pigmentation.

### Concluding remarks.

Transcriptional divergence of key morphogenic genes has been hypothesized to play an important role in the morphological innovations in eukaryotes. Our study demonstrates that rewiring of transcription for morphogenic genes in different fungal lineages has contributed to successful perithecial development and supports the previous findings that changes in gene expression levels of homologous genes for developmental processes are as important as emergence of novel genes that are incorporated into the existing gene regulatory network. About 40% of annotated genes in 2,000 fungal genomes listed in the JGI website (https://mycocosm.jgi.doe.gov/mycocosm/home) encode hypothetical proteins with unknown function. This fact urges a community-level effort for functional characterization of these conserved unknowns. Our approach to finding conserved unknowns with evolved changes in gene expression proved efficient for discovering novel gene function. Dissecting molecular function of these hypothetical proteins will shed light on novel mechanisms by which different lineages of fungi achieve successful sexual development according to their evolutionary contexts.

## MATERIALS AND METHODS

### Genomic and transcriptomic data.

The genome sequence of the F. graminearum PH-1 strain (NRRL 31084) was used throughout this study (NCBI genome assembly accession no. GCA_000240135.3; Ensembl annotation v.32) (79). For *F. neocosmosporiellum*, the genome sequence of *F. neocosmosporiellum* NRRL 22166 strain was used (GCA_006518225.1; the gene annotation file can be found in NCBI GEO through GSE124553 (44)). For M. oryzae, the genome sequence of the Pyricularia oryzae 70-15 strain was used (GCA_000002495.2; Broad Institute annotation MG8). For N. crassa, the genome sequence of OR74A strain was used (GCA_000182925.2; Broad Institute annotation NC12). For *C. globosum*, the genome-sequenced strain (CBS 148.51) was used (GCA_000143365.1; Joint Genome Institute annotation v1.0).

To obtain transcriptome data for M. oryzae during perithecial development, we crossed two fertile strains with opposite mating types, 4091-5-8 (*MAT1-2*) and 4136-4-3 (*MAT1-1*) (46), on carrot agar, a medium that have been commonly used for the other four species to minimize influence of external environmental conditions. Conidia of strain 4091-5-8 (1 × 10^4^ spores/mL) were spread on carrot agar in a petri dish (6 cm in diameter) and then grown for 8 days. Hyphae and conidia were gently scraped away from the surface of the 8-day-old culture. The culture was washed with sterile water a couple of times and air-dried, and then 1 mL of conidial suspension of strain 4136-4-3 (1 × 10^5^ spores/mL in 2.5% Tween 60) were spread onto the surface of the culture. After 6 days of the mixture of the two strains, seta formation on heavily melanized stroma of the 4091-5-8 strain was observed, which is a sign of ascogonium formation beneath the agar surface (80). Fungal tissues representing each developmental stage were collected by scraping the surface of fungal colonies around setae with a razor blade and subjected to RNA extraction for transcriptome analysis: S0, 24 h before formation of setae; S1, when seta formation is evident; S2, 24 h after seta formation; S3, 48 h after seta formation, when paraphyses were observed in a squashed mount of perithecia; S4, 72 h after seta formation, when asci were observed in a squashed mount of perithecia; S5, 120 h after seta formation, when asci were filled with ascospores. Fungal tissues collected from a single plate were considered a biological replicate. Total RNA was extracted as previously described (44), and RNA quality was confirmed using an Agilent 2100 Bioanalyzer (Agilent Technologies, Palo Alto, CA, USA). Total RNA from three biological replicates of each developmental stage were pooled, and 2 μg of total RNA was used for cDNA library construction using the KAPA Stranded RNA-Seq library preparation kit (Kapa Biosystems, Wilmington, MA, USA). Samples were run on an Illumina HiSeq 2500 platform (Illumina, Inc.; single-end 50-bp reads) at Michigan State University’s Research Technology Support Facility. The raw data of RNA-seq reads were processed as previously described (44), and filtered reads were mapped to the genome sequence of *P. oryzae* 70-15 strain, using the HISAT2 program v2.0.4 (81). Then, we tallied mapped reads on genomic features, using the htseq-count program in the HTSeq package v0.6.1 (82). On average, 83% of mapped reads were overlapped to exons in the MG8 annotation. Gene expression levels in RPKM values were computed and normalized by effective library size estimated by trimmed mean of M values, using the edgeR R package v3.14.0 (83).

### CACE.

For continuous ancestral character estimation (CACE), single-copy ortholog clusters of protein sequences deduced from the five sordariomycete genomes were identified, using the ReMark (84) and OrthoMCL (85) programs, specifying inflation factors of 1.6 and 2.5, respectively. The predicted SCOGs from the two programs were combined, resulting in a total of 3,942 SCOGs. We excluded SCOGs including genes that exhibited basal expression levels throughout perithecial development because RNA-seq data are inherently noisy, and small fluctuations of lowly expressed genes in their expression levels between developmental stages could be overestimated in calculation of fold changes that will be used for prioritizing candidate genes for knockout studies in our downstream analysis. RPKM values for genes annotated in the five sordariomycete genomes were transformed by *Z*-normalization, using the zFPKM R package v1.6.0 (86), and genes with averaged *Z*-normalized RPKM values greater than or equal to −3 were considered active genes in the perithecial transcriptome data, according to Hart et al. (47). About 8 to 14% of predicted genes in the five species have averaged *Z*-normalized RPKM values smaller than −3. Seventy-eight SCOGs including these lowly expressed genes were excluded in the downstream analysis.

Tallies of expression such as RPKM are not considered comparable between orthologs (48). Therefore, based on a rationale explained in detail elsewhere (10), relative gene expression levels across all developmental stages for genes in each of the five species were estimated using LOX (87). We then calculated the fold change of relative expression levels between each adjacent pair of developmental stages. The fold change between developmental stages and the phylogeny of the five species in Newick tree format were supplied as input files to an in-house R program that utilizes the R package APE v5.3 (88, 89), which provided ancestral changes in expression across adjacent stages at all internal nodes for every SCOG.

### Functional investigation of top-ranked genes in CACE analyses.

To generate a priority list of genes whose expression has significantly increased over the evolutionary processes, fold changes between adjacent pair of developmental stages in F. graminearum, M. oryzae, or N. crassa were subtracted from the estimated fold changes in the MRCAs of the five species, sorting the 3,864 SCOGs by their largest difference. The top 25 genes ranked in the list were investigated by generating gene deletion mutants from the F. graminearum PH-1 wild-type strain. For M. oryzae, 10 hypothetical genes ranked in the top 25 were individually deleted in two highly fertile wild-type strains, 4091-5-8 and 4136-4-3. In addition, several genes in F. graminearum were selected for functional studies from a priority list where fold changes in the MRCA of the five species were contrasted with fold changes in a common ancestor of F. graminearum and *F. neocosmosporiellum* that belongs to the family Hypocreaceae.

To generate gene deletion mutants in F. graminearum and M. oryzae, we used a split marker strategy (90), in which the left and right flanking regions of the target gene were amplified and merged with a minimal gene cassette encoding hygromycin phosphotransferase (*hph*) under the control of the *trpC* promoter from Aspergillus nidulans. Fusion PCR was performed as described previously (91), and primers used in targeted gene deletion are listed in Table S2 in the supplemental material. In brief, left flanking regions and right flanking regions of the coding sequence were amplified, using L5 and L3 primer pairs and R5 and R3 primer pairs, respectively. L3 and R5 primers have 27-nucleotide (nt)-long overhang sequences complementary to the 5′ and 3′ ends of the minimal *hph* cassette (1,376 bp), which was amplified from pCB1004 plasmid (92), using HYG5 and HYG5 primers. Then, the PCR amplicons were fused by overlap extension, in the order of left flanking region, *hph* cassette, and right flanking region. Finally, two split marker constructs were amplified from the fused amplicon, using nested primer pairs (N5 and HY-R primers for the left-half construct and YG-F and N3 primers for the right-half construct).

10.1128/mbio.01100-22.7 TABLE S2 Primers used in this study. Download Table S2, DOCX file, 0.02 MB. Copyright © 2022 Kim et al. 2022 Kim et al. https://creativecommons.org/licenses/by/4.0/ This content is distributed under the terms of the Creative Commons Attribution 4.0 International license.

The two split marker constructs were then pooled and introduced by polyethylene glycol-mediated transformation of protoplasts (93). Following transformation, transformants resistant to hygromycin (150 μg/mL for F. graminearum and 450 μg/mL for M. oryzae) were examined for deletion of the target gene by diagnostic PCR checks in which L5 and R3 primers outside the area of gene replacement were used to amplify the entire region, documenting the gene replacement by a shift in size (Fig. S4). In case the shift in size was not big enough to confirm target gene replacement with the *hph* cassette, the presence of the *hph* cassette in the targeted locus was verified by PCR checks with two primer pairs: L5 and HY-R primers for upstream region check (UR), and YG-F and R3 primers for the downstream region check (DR) (Fig. S4). Perithecial formation in knockout mutants was examined by stereomicroscopy for size and number formed, as well as the degree of ascospore release at maturity (cirrhus production and spore discharge). Squash mounts of young developing perithecia in water were examined using a compound microscope to ascertain the presence of paraphyses or asci as well as morphology and maturity of ascospores.

10.1128/mbio.01100-22.4 FIG S4 Confirmation of the gene deletions by PCR. Download FIG S4, PDF file, 0.5 MB. Copyright © 2022 Kim et al. 2022 Kim et al. https://creativecommons.org/licenses/by/4.0/ This content is distributed under the terms of the Creative Commons Attribution 4.0 International license.

For genetic complementation, pDS23 plasmids (94) harboring one of the prioritized genes whose knockouts exhibited defective perithecial phenotypes were transformed into protoplasts of knockout mutants lacking the prioritized gene. Following transformation, transformants resistant to nourseothricin (200 μg/mL; Jena Bioscience, Jena, Germany) were examined for reintroduction of the target genes (FGRRES_06285 and -_6797) by diagnostic PCR checks, and the successful recovery of defective perithecial phenotypes observed in knockouts of FGRRES_06285 and -_6797 was confirmed in the strains harboring the pDS23 plasmids (Fig. S5).

10.1128/mbio.01100-22.5 FIG S5 Genetic complementation of knockout mutants arrested at a protoperithecial stage. Download FIG S5, PDF file, 0.3 MB. Copyright © 2022 Kim et al. 2022 Kim et al. https://creativecommons.org/licenses/by/4.0/ This content is distributed under the terms of the Creative Commons Attribution 4.0 International license.

### Estimation of expression divergence for single-copy orthologs.

To classify SCOGs into “functional SCOGs” and “hypothetical SCOGs,” protein domains were predicted for all the gene models in the five species, using the InterProScan program. Genes harboring one or more predicted protein domains were assigned to be “functional genes” (E value, <10^−10^). For the expressed 3,864 SCOGs in the five species, if “functional genes” are found in more than two species in a SCOG cluster, corresponding SCOGs were classified as “functional SCOGs,” otherwise as “hypothetical SCOGs.” To estimate degrees of expression divergence for SCOGs, relative expression levels of genes across the six developmental stages were calculated from RPKM values, summing up to 1. Pairwise correlations of relative gene expression levels in a SCOG cluster between the five species were measured as Euclidean distance. The Euclidean distance values between species for SCOGs were averaged. Probability density plots of the averaged Euclidean distance for SCOGs were displayed separately for “functional SCOGs” and “hypothetical SCOGs.”

### Genomic phylostratigraphy.

Genomic phylostratigraphy for the N. crassa genome was estimated as previously described (95). Briefly, we used the SIMAP (Similarity Matrix of Proteins) database developed by MIPS (https://cube.univie.ac.at/resources/simap) and calculated the pairwise similarity of protein-coding sequences by Smith-Waterman pairwise comparison (96, 97). Homologous proteins in hierarchical taxonomic units for the MIPS-curated N. crassa proteins were retrieved from the database. The genes were classified into mutually exclusive groups ranked in association with their phylostratigraphy, including Euk/Prok-core, Dikarya-core, Ascomycota-core, Pezizomycotina-specific, Neurospora-orphans, and others (not classified). To ensure quality of the retrieved data set, homologous sequences of protein-coding genes in N. crassa were searched in genomes of *C. globosum*, Saccharomyces cerevisiae, Phanerochaete chrysosporium, Drosophila melanogaster, and Arabidopsis thaliana, respectively, using the same criteria used for the SIMAP database. Less than 1% of the SIMAP data and our homology search data showed discrepancies in terms of groupings. We then used ortholog groups recognized in the FungiDB database (http://Fungidb.org) to identify orthologs shared and missed in genomes of N. crassa, Magnaporthe grisea, Trichoderma asperellum, and F. graminearum to reconstruct the phylostratigraphy of the F. graminearum genome and to identify the Hypocreales lineage-specific ortholog groups.

### Accession number(s).

The RNA-seq data generated in the present work have been deposited in NCBI’s Sequence Read Archive and are accessible through SRA accession no. SAMN23838533 to SAMN23838538 and BioProject accession no. PRJNA787662.
