# Supplementary material for: Transcriptional Divergence Underpinning Sexual Development in the Fungal Class Sordariomycetes
Source: mBio. 2022 May 31;13(3):e01100-22. doi: 10.1128/mbio.01100-22 (PMC9239162; doi:10.1128/mbio.01100-22)
Supplement: FIG S1 [file mbio.01100-22-s0002.pdf]

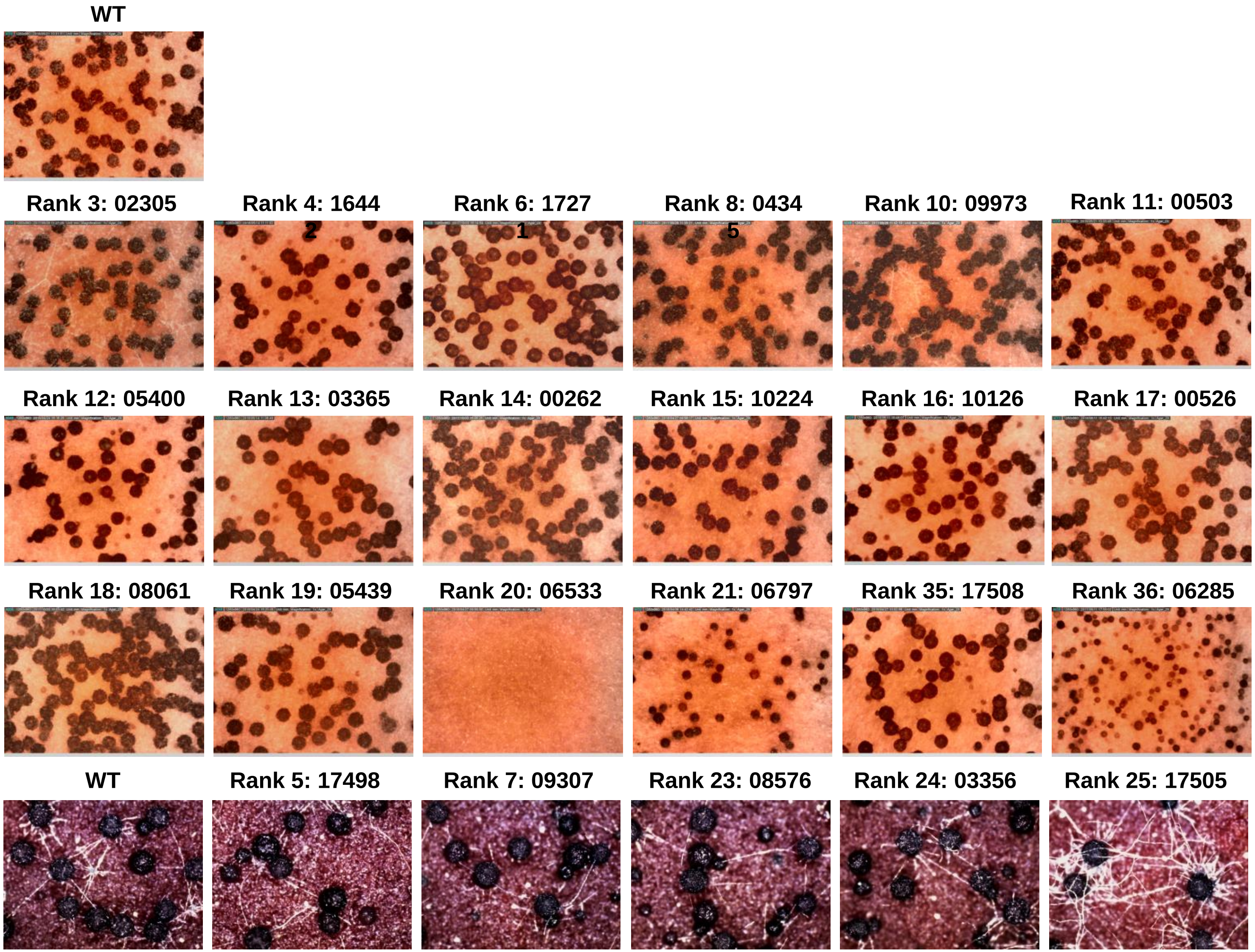

Fig. S1. Perithecial morphology of knockout mutants for top 25 ranked genes. Perithecia (black spheres) formed on carrot agar. Photos taken 7 days after sexual induction. wild-type strain PH-1, WT. Five digit numbers indicate gene id coming after the FGRRES epithet.
