# Supplementary material for: Transcriptional Divergence Underpinning Sexual Development in the Fungal Class Sordariomycetes
Source: mBio. 2022 May 31;13(3):e01100-22. doi: 10.1128/mbio.01100-22 (PMC9239162; doi:10.1128/mbio.01100-22)
Supplement: FIG S2 [file mbio.01100-22-s0003.pdf]

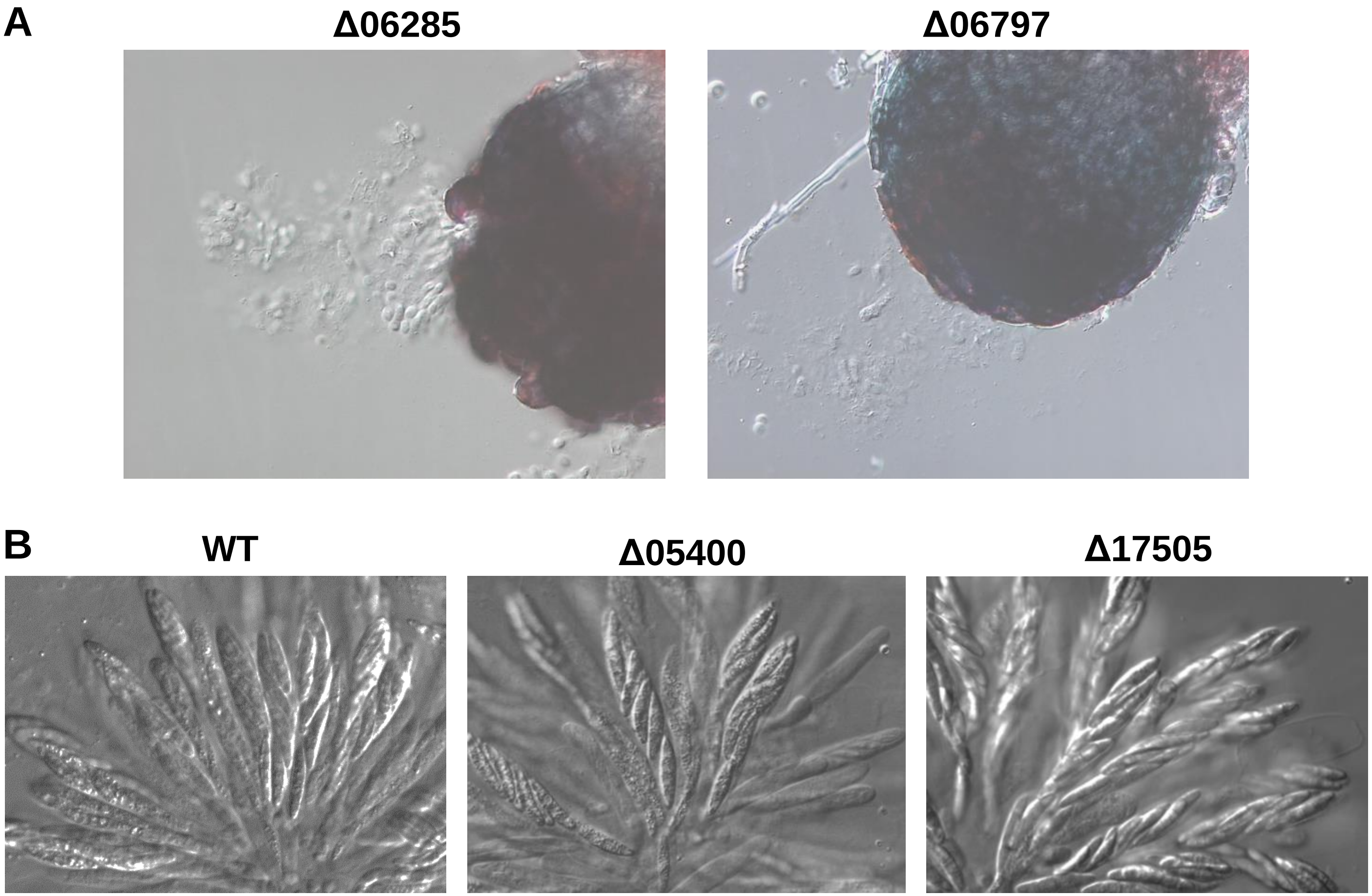

Fig. S2. Sexual phenotypes of novel genes encoding hypothetical proteins and hydrolytic enzymes. (**A**) a squashed protoperithecium of knockout mutants of FGRRES_06285 and _06797, showing no asci and ascospore production. (**B**) Normal asci and ascospores production in knockout mutants of FGRRES_05400 and _17505.
