# Supplementary material for: Transcriptional Divergence Underpinning Sexual Development in the Fungal Class Sordariomycetes
Source: mBio. 2022 May 31;13(3):e01100-22. doi: 10.1128/mbio.01100-22 (PMC9239162; doi:10.1128/mbio.01100-22)
Supplement: FIG S3 [file mbio.01100-22-s0004.pdf]

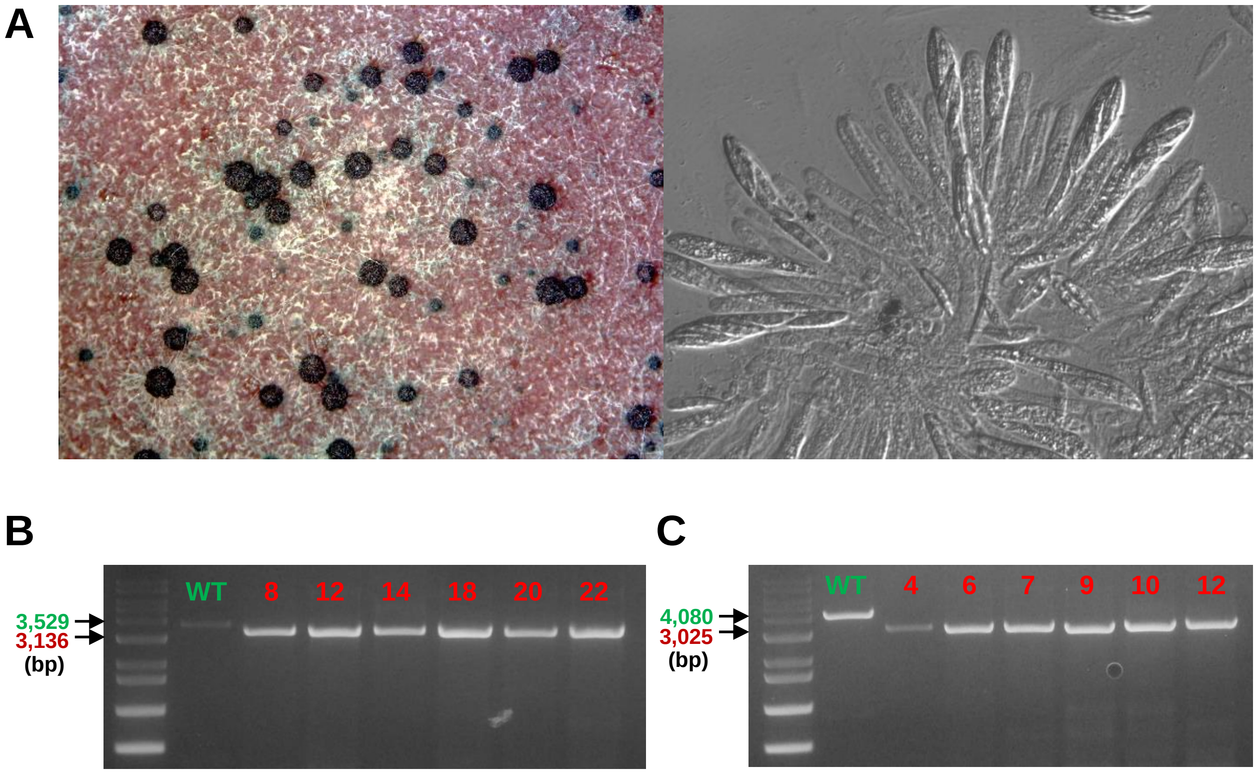

Fig. S3. Sexual phenotypes of FGRRES_06775 homologous to NCU00309. (**A**) Normal perithecia (left panel) and asci (right panel) production in a knockout mutant of FGRRES_06775. The authenticity of knockout mutants of FGRRES_06775 (**B**) and FGRRES_00193 (**C**) was checked in PCR analysis along with their wild-type progenitor (WT), using L5 and R3 primers that distinguish knockouts from the WT progenitor. Numbers in red indicate independent knockout mutants obtained by genetic transformation. The expected sizes of PCR amplicons for WT and knockout mutant (KO) were depicted by arrows.
