# Supplementary material for: Transcriptional Divergence Underpinning Sexual Development in the Fungal Class Sordariomycetes
Source: mBio. 2022 May 31;13(3):e01100-22. doi: 10.1128/mbio.01100-22 (PMC9239162; doi:10.1128/mbio.01100-22)
Supplement: TABLE S2 [file mbio.01100-22-s0008.docx]

**Table S2.** Primers used in this study.

| **Gene ID** | **Primer** | **Sequence (5'-3')** |
| --- | --- | --- |
| For *hph* amplification | HYG5 | GCTTGGCTGGAGCTAGTGGAG |
|  | HYG3 | CGGTCGGCATCTACTCTATTCCTT |
| For 3^rd^ round amplification | YG-F | CGATGTAGGAGGGCGTGGATATGTC |
|  | HY-R | TGTAGTGTATTGACCGATTCCTTGCG |
| FGRRES_02305 | 02305L5 | GAGCAGGAAGTTGAGAATGAAATTCC |
|  | 02305L3 | GTTGACCTCCACTAGCTCCAGCCAAGCGGTCCCCATTGTATCGTCAATATG |
|  | 02305R5 | GGCAAAGGAATAGAGTAGATGCCGACCGAAGACAAAGAAGCTAGATAAGAGGG |
|  | 02305R3 | GCGACATGTTGTCAGAAGCAAGG |
|  | 02305N5 | GAGGAAAGCGTTATCTGAGTGGGC |
|  | 02305N3 | CGCCGTGATCCGTCGTTAACATG |
| FGRRES_16442 | 16442L5 | GTCGAGACTGGATGTCCTGCTTG |
|  | 16442L3 | GTTGACCTCCACTAGCTCCAGCCAAGCGCGGAGAGCCATGATGTAAGAGAAG |
|  | 16442R5 | GGCAAAGGAATAGAGTAGATGCCGACCGCGGAGGTGAGTGTATGAGTGGTACAG |
|  | 16442R3 | CTTCGGCTACTACGAGACCATCTG |
|  | 16442N5 | CGGATAGAGTGAGGAATGAAGTCACG |
|  | 16442N3 | CGGTGCACACTCACATGACAAACAC |
| FGRRES_17498 | 17498L5 | CGACAATGATCGGCGTAGATACC |
|  | 17498L3 | GTTGACCTCCACTAGCTCCAGCCAAGCGCCAAGTCCGCATATTCGATCTC |
|  | 17498R5 | GGCAAAGGAATAGAGTAGATGCCGACCGCTTGAGTGATGGGAAGGCATTGAC |
|  | 17498R3 | CTGTAATAACGAAGGCGACGCAAG |
|  | 17498N5 | GATCTTCCAAGACACCGATCAGC |
|  | 17498N3 | CCGGGTCATTGCTTGTAATACTACC |
| FGRRES_17271 | 17271L5 | GGCATTGATTTAAGCATCATTCTTGCC |
|  | 17271L3 | GTTGACCTCCACTAGCTCCAGCCAAGCGCCGTGTTCTGTTCCATGATCGTC |
|  | 17271R5 | GGCAAAGGAATAGAGTAGATGCCGACCGGAAGGAAGTCAAGAAGGATGCCTAAG |
|  | 17271R3 | CCAAGCATGGATTGATTCATGGCG |
|  | 17271N5 | CGATACATCCTTCCCTCTGCACC |
|  | 17271N3 | GAAGAAGGCGAGGAGACCCTG |
| FGRRES_09307 | 09307L5 | GCTCCAACGCTTCGATCTTAATGTC |
|  | 09307L3 | GTTGACCTCCACTAGCTCCAGCCAAGCGGACAACATCAGGCATAAAGCACC |
|  | 09307R5 | GGCAAAGGAATAGAGTAGATGCCGACCGGCTACGGCATGGGAAGTAGCTTAC |
|  | 09307R3 | GGTAGAATGGATTCGCGACAGTG |
|  | 09307N5 | CTCGACAACATAGTAGGCGTCATG |
|  | 09307N3 | GAATAAGGCTATCGCCGGTAGAAC |
| FGRRES_04345 | 04345L5 | GGCTGTTGTTCTCCGCAACGC |
|  | 04345L3 | GTTGACCTCCACTAGCTCCAGCCAAGCGTTGGCGATGTGTAGGTGAAAGAG |
|  | 04345R5 | GGCAAAGGAATAGAGTAGATGCCGACCGCCAACGCTGCCAGGTGTCGTC |
|  | 04345R3 | GATCGCTTGCTAGGTCACGTACTG |
|  | 04345N5 | GACAACCTGACAACCAGCAAGG |
|  | 04345N3 | GATTCGCTCGCTTGAGTGACGAG |
| FGRRES_09973 | 09973L5 | GGTAGGTAGTCAGTCAAGGCTGC |
|  | 09973L3 | GTTGACCTCCACTAGCTCCAGCCAAGCAGACATCCTGCCGTCTGTTGAGTG |
|  | 09973R5 | GGCAAAGGAATAGAGTAGATGCCGACCGGACTATCCAGAAGAATCTGAGAGGG |
|  | 09973R3 | CTGATATTGGGTCCAAGACTGGTC |
|  | 09973N5 | GAATTCACCTGCAACATACGCTTGG |
|  | 09973N3 | GGACTTGTACGAAATCACCCTCATC |
| FGRRES_00503 | 00503L5 | GGAATTCGATCTCGCAAGGTTGTC |
|  | 00503L3 | GTTGACCTCCACTAGCTCCAGCCAAGCGCAGCATAGTGTGTGGCTACGAAG |
|  | 00503R5 | GGCAAAGGAATAGAGTAGATGCCGACCGCTGTCATTACCAGGCTCTCCATTC |
|  | 00503R3 | CTGAGTTCAACGAAGTGATGAGGG |
|  | 00503N5 | GGGAAGTAGTCATGATAAGCACGG |
|  | 00503N3 | GTGATGCTGCGAGACATATTGTCAATG |
| FGRRES_05400 | 05400L5 | GTTGGACTCGTCTATCTCCTTTGC |
|  | 05400L3 | GTTGACCTCCACTAGCTCCAGCCAAGCGAACTGTGAGGATAGCCTCTTGTG |
|  | 05400R5 | GGCAAAGGAATAGAGTAGATGCCGACCGACCACTGGGCATGACTGAAGTTAG |
|  | 05400R3 | CGTACATGCGTGACTTTTCCAGC |
|  | 05400N5 | CGAGTTTTGTCTGCTGATAGGCAC |
|  | 05400N3 | CAGGATTCAACAGAAACGTGAGAGG |
| FGRRES_03365 | 03365L5 | CCAAGAGGTTGTGCTATGGAGATTCC |
|  | 03365L3 | GTTGACCTCCACTAGCTCCAGCCAAGCCGAATGGACATGATGACTTGCTTTGC |
|  | 03365R5 | GGCAAAGGAATAGAGTAGATGCCGACCGGCTCTTAGTTGGTGGAGTGAAGAGG |
|  | 03365R3 | GGTTCCAGCAGATGAACGATTTGAC |
|  | 03365N5 | GTGATAGAGGGTTCTTCGCGGCAG |
|  | 03365N3 | GAAGGCTACGAATGGCACAACGC |
| FGRRES_00262 | 00262L5 | GATATCGTCGGCAATCTGCTGACG |
|  | 00262L3 | GTTGACCTCCACTAGCTCCAGCCAAGCGCTGTTGGGCATCGATCATCACAAG |
|  | 00262R5 | GGCAAAGGAATAGAGTAGATGCCGACCGGCAAGAGAAGCAAGTGGCGGATTAG |
|  | 00262R3 | CCTGTCTCTGACGTTCCTGATATTCC |
|  | 00262N5 | GATGGAAGAAGGTGGTCTCGACCTG |
|  | 00262N3 | CAGAGCCTCCTGCTGAAACTACC |
| FGRRES_10224 | 10224L5 | GGATTACAGACTCGGCACAGGATG |
|  | 10224L3 | GTTGACCTCCACTAGCTCCAGCCAAGCGATTCCTTCCAACGGCGAGTTCCC |
|  | 10224R5 | GGCAAAGGAATAGAGTAGATGCCGACCGGGACCGATTGGGACAATATACAGTC |
|  | 10224R3 | AGTTTAGCGACCTAGCCAGCGAC |
|  | 10224N5 | GGAGGAGGGATGGTTGAACTATG |
|  | 10224N3 | CGACTTTCCCAACAAAGACAACGAC |
| FGRRES_10126 | 10126L5 | CCTGAAAGTTCGGTGAGTCCATCAG |
|  | 10126L3 | GTTGACCTCCACTAGCTCCAGCCAAGCCTATGTTGTCAGGCATACTTGGCAG |
|  | 10126R5 | GGCAAAGGAATAGAGTAGATGCCGACCGGAAATGCTGAGGATGCCATGGTCAAG |
|  | 10126R3 | CGTCATCTACATCAACGGGAGTACG |
|  | 10126N5 | GCTCACCGACAAGACATTGCTATC |
|  | 10126N3 | CAGATCTCAAGCTGAAGCAGGTCG |
| FGRRES_00526 | 00526L5 | GGATCAATCAGGATTGCGTGAACCG |
|  | 00526L3 | GTTGACCTCCACTAGCTCCAGCCAAGCGAGGAGACGAGAGGAGTTTCTTTGCC |
|  | 00526R5 | GGCAAAGGAATAGAGTAGATGCCGACCGCCACCAAGAGCCATGATAGCAAGTC |
|  | 00526R3 | GGCAGCGCAGATACTCTTCCATTC |
|  | 00526N5 | GCAGGTTTATCGCAACGCTCCATC |
|  | 00526N3 | CACGCCAGACGAAGAATACCACATC |
| FGRRES_08061 | 08061L5 | CATTGTCAACACAGCAAGCTGCTAC |
|  | 08061L3 | GTTGACCTCCACTAGCTCCAGCCAAGCGGCCAAGGGGTAAAGACAAGACTC |
|  | 08061R5 | GGCAAAGGAATAGAGTAGATGCCGACCGGCCCGATATCTATGGCGCTATCC |
|  | 08061R3 | GTCACAGGAGACAGACAGGAAGC |
|  | 08061N5 | CAACCTGACTATCGTCTGCTTTGC |
|  | 08061N3 | CGCCGGACCCAAATGGTAAACAG |
| FGRRES_05439 | 05439L5 | CAGCACTGGGAGAAAGAGAGACAG |
|  | 05439L3 | GTTGACCTCCACTAGCTCCAGCCAAGCGGCAACGAGCTTCTTCTCGTATGAG |
|  | 05439R5 | GGCAAAGGAATAGAGTAGATGCCGACCGGATAGTGGAACGGAAGCTGGAAATTG |
|  | 05439R3 | GTGACATATTTCGGGAGCTGACAG |
|  | 05439N5 | CACCACTACCCGATACTAAAGAGCC |
|  | 05439N3 | TGTGACGGTGTTGTAGATGTCGTG |
| FGRRES_06533 | 06533L5 | CTGTCACCAAGACATGCAGATGGC |
|  | 06533L3 | GTTGACCTCCACTAGCTCCAGCCAAGCCGACTTGATGATAGTACACAGCCCAG |
|  | 06533R5 | GGCAAAGGAATAGAGTAGATGCCGACCGAGACGGCTATAGATTCAGGATAGACG |
|  | 06533R3 | GTCCGACACTCCAACTCCAAGAAC |
|  | 06533N5 | CACCGTTTGACAAGTCGTCCCAC |
|  | 06533N3 | CACCGAAAAGCTCAAGCACTACATG |
| FGRRES_06797 | 06797L5 | CTCTAGCATGGCACCCTACTGAAG |
|  | 06797L3 | GTTGACCTCCACTAGCTCCAGCCAAGCGCGAATGTCAATGGAACGAATCAGT |
|  | 06797R5 | GGCAAAGGAATAGAGTAGATGCCGACCGCGAAGACGAAGACGAGTGACGATG |
|  | 06797R3 | CTGTCTCTCATCTTTGCCCCTGTC |
|  | 06797N5 | GACCGAAGAATCATCTTCTGGGAC |
|  | 06797N3 | ACATACTGTACGAGTCTGACATTGTC |
| FGRRES_08576 | 08579L5 | GGTGTTAACGAGATGCCATAACC |
|  | 08579L3 | GTTGACCTCCACTAGCTCCAGCCAAGCCTCGGTGAAGTAGAAGCGTTATGAG |
|  | 08579R5 | GGCAAAGGAATAGAGTAGATGCCGACCGGTACTATCCGCATCGGACTTACAGC |
|  | 08579R3 | GGAGTGTCCTTCTGGACAATGTAAG |
|  | 08579N5 | GGAGATCCGTTGGTATATTCTGAGC |
|  | 08579N3 | GATACACACCTTCCTCCTCTCG |
| FGRRES_03356 | 03356L5 | CGATCAGTTCACATCCGACTCTATC |
|  | 03356L3 | GTTGACCTCCACTAGCTCCAGCCAAGCGTTGTGGTTATGTGGGCGAGTTAC |
|  | 03356R5 | GGCAAAGGAATAGAGTAGATGCCGACCGGTAGCTGATTCGGTGGTTCGTTG |
|  | 03356R3 | CACTAGCAACCTGGTACCACTGC |
|  | 03356N5 | GCTCGTCTTCTGCTCGAAGTAATC |
|  | 03356N3 | CGAACAGAAGCCATTCGACAAC |
| FGRRES_17505 | 17505L5 | GAGGATTACGTTACGTGCCTTGG |
|  | 17505L3 | GTTGACCTCCACTAGCTCCAGCCAAGCCAAATGGCGTCGAATCTGCACTG |
|  | 17505R5 | GGCAAAGGAATAGAGTAGATGCCGACCGGCTGTTGGATTGACAGGAGGAGTC |
|  | 17505R3 | CATCGAATACGAGCTTCGTCTCAG |
|  | 17505N5 | CAGCTTGTAGATGAGGCTTGAAC |
|  | 17505N3 | CTGTCATGAGGGCCAGAAGGTG |
| FGRRES_17508 | 06285L5 | GCACATTGTGACACTGCTGTATCC |
|  | 06285L3 | GCCAAGCCCGATGCCGTTTTTGCGTAGCG |
|  | 06285R5 | ATGCCGACCAAGGCGGAGGCACGAGAAAAG |
|  | 06285R3 | CAACTTTCCCGGAGAGTACAAGAC |
|  | 06285H5 | AACGGCATCGGGCTTGGCTGGAGCTAGTG |
|  | 06285H3 | CTCCGCCTTGGTCGGCATCTACTCTATTCCTTTG |
|  | 06285N5 | GGCAGTGTAGATAAAGAGCGGC |
|  | 06285N3 | GAACCTCGGTCTATTCAAGGCATG |
| FGRRES_06285 | 17508L5 | CACTCATCCCAAGTGTCGCGTTG |
|  | 17508L3 | GTTGACCTCCACTAGCTCCAGCCAAGCCTTGACTATGTCCAAGTTCCGGTC |
|  | 17508R5 | GGCAAAGGAATAGAGTAGATGCCGACCGGAACAGCCTCATCTAAGCAGTACC |
|  | 17508R3 | GACGCATTCATGCAATGCCTGTC |
|  | 17508N5 | CTTGGCCTTCATCTCTCCTCATC |
|  | 17508N3 | CAAACCCACCGCTAGGATATCTC |
| FGRRES_06775 | 06775L5 | GATGTGGCGCTAATGTTGTAGCC |
|  | 06775L3 | GTTGACCTCCACTAGCTCCAGCCAAGCCTCTAGGACTCTGAGCACTGGAAAGC |
|  | 06775R5 | GGCAAAGGAATAGAGTAGATGCCGACCGCTCTGCATTCTTATCAGAGCACCTC |
|  | 06775R3 | GTCAGGATCTCAGCAGCCTCATG |
|  | 06775N5 | CATAGTCTTACTGCGCTAGAGACC |
|  | 06775N3 | GAGGTGATTCGAGATGTCGTTGTC |
| FGRRES_00193 | 00193L5 | GTTGAGCAGGAGGTCGAGGTTG |
|  | 00193L3 | GTTGACCTCCACTAGCTCCAGCCAAGCGTTGCGCCGAAGCATCTCTCATG |
|  | 00193R5 | GGCAAAGGAATAGAGTAGATGCCGACCGCACGAACAATGCTGGAGAAGCCAG |
|  | 00193R3 | CGACACTGTCGCCGAGATAGTAC |
|  | 00193N5 | CACTGGCGCTAAGCAGCTTG |
|  | 00193N3 | GTACGCGACAAAGTCGGCGC |
| FGRRES_06797complementation | pDS06797_fwd | CTAACAGCTACAGATCATGTCTTCCAACCCACCTCC |
|  | pDS06797_rev | TGCTCACCATAAGCTCCTCGTCTTCGTCTTCGTCTTC |
| FGRRES_06797amplification | 06797RT5 | CGAATGTGGGTTGACAGCAGATCG |
|  | 06797RT3 | GAGAGGAACATTGTACGCCTTGGG |
| FGRRES_06285complementation | 06285Avr5 | CCTAGGGAGCTGGAATTGCACTTTCTGCTGC |
|  | 06285Bgl3 | AGATCTAGCGTTAAGGTCCAGGTCCTCC |
| MGG_03672 | MG03672L5 | GAAGGGAGTTCCGTACTGGTTGG |
|  | MG03672L3 | GTTGACCTCCACTAGCTCCAGCCAAGCCTAGTGCTGAGGACCTAACAGGAAAC |
|  | MG03672R5 | GGCAAAGGAATAGAGTAGATGCCGACCGCAAGCAGAAGCTTTGCTGCTCTAGG |
|  | MG03672R3 | GTCACGATGTTCGGAGTGATGTTGC |
|  | MG03672N5 | CGTTGGATGAGGATCACAAGTGGTG |
|  | MG03672N3 | GAGTTTTGACGACATGGTGCCCAC |
| MGG_03403 | MG03403L5 | GACGAGAAGCAGCAGGTGGTTTAC |
|  | MG03403L3 | GTTGACCTCCACTAGCTCCAGCCAAGCCCAGGGAGTAATAAGTAGGTAGCGTC |
|  | MG03403R5 | GGCAAAGGAATAGAGTAGATGCCGACCGGGAGTTTATAGCCTTGGTGTAAGCGAC |
|  | MG03403R3 | GGCGAATGAGAACGAGAGTTGAGAC |
|  | MG03403N5 | GTTCGGACACCATGAGCACAGTG |
|  | MG03403N3 | GACAGAGAGGGAAACTAAAGCAAGC |
| MGG_02581 | MG02581L5 | GGAATTGCCATCTCAGGCCAAATG |
|  | MG02581L3 | GTTGACCTCCACTAGCTCCAGCCAAGCGTTATGGTTAAGGCTACTGAGCTGGG |
|  | MG02581R5 | GGCAAAGGAATAGAGTAGATGCCGACCGGCCGTTTATTACTGCGTGTCCACC |
|  | MG02581R3 | CATCATTTGCGGCAGACACGGTAG |
|  | MG02581N5 | CACTCCGTCGGAAGATGTTGATACC |
|  | MG02581N3 | GTGAGCCGCATACTACAGATATGGC |
| MGG_09879 | MG09879L5 | GGTATAACGGTTGATAACCCGGC |
|  | MG09879L3 | GTTGACCTCCACTAGCTCCAGCCAAGCGCCTAAGATAACTTCCATTCCAAGGCC |
|  | MG09879R5 | GGCAAAGGAATAGAGTAGATGCCGACCGGGACCAGCAATAGAGGATGGTAATGC |
|  | MG09879R3 | GCATATCAGGATCTGGTTGACCGAG |
|  | MG09879N5 | CCTGTACAGTGCGTGCACAGTG |
|  | MG09879N3 | GCAGTCGGTTAATCGTACCCAGG |
| MGG_16792 | MG16792L5 | GTATCTTCCGTAACGACGTAGGCG |
|  | MG16792L3 | GTTGACCTCCACTAGCTCCAGCCAAGCAGCATGGAATGGAGATGTTTCCTGTG |
|  | MG16792R5 | GGCAAAGGAATAGAGTAGATGCCGACCGCCATTGCTGGCATGATGGTCATACC |
|  | MG16792R3 | CGATTTCGCAAAGGATCGGCGTTC |
|  | MG16792N5 | GTGCAGGGTAGGTAGGTACTCAGG |
|  | MG16792N3 | GACGATTTGCCTGCCGGAG |
| MGG_09418 | MG09418L5 | GGTATTGAGATCCTTGGCGAGAGG |
|  | MG09418L3 | GTTGACCTCCACTAGCTCCAGCCAAGCCGTGTCGATGCCTTGTTCGTTATTG |
|  | MG09418R5 | GGCAAAGGAATAGAGTAGATGCCGACCGGCATCATGAAATGGCGGAGGAGTTG |
|  | MG09418R3 | CGATCATCCGGGGTGATTGACAAG |
|  | MG09418N5 | CTCATGCTTCACGCCAATCTGGATC |
|  | MG09418N3 | GATCTCCGAATGCAGGCCAACAG |
| MGG_17975 | MG17975L5 | CAGAGACATTCTTGTGGATTCCG |
|  | MG17975L3 | GTTGACCTCCACTAGCTCCAGCCAAGCCCAGATGTTTGTCCACAGAGAGTG |
|  | MG17975R5 | GGCAAAGGAATAGAGTAGATGCCGACCGGATATCCCGAGGTCTATGCCATG |
|  | MG17975R3 | GGCAAGTTTGAGGACGAGATGC |
|  | MG17975N5 | GACTTTCAGCTCTCGGACGGAC |
|  | MG17975N3 | GCTCGATAAGGGAAGGGATGG |
| MGG_09955 | MG09955L5 | GTGGGTCAACTAGTCTAGATACCTACC |
|  | MG09955L3 | GTTGACCTCCACTAGCTCCAGCCAAGCGACTGACTTGTCACCCTGCCACTTC |
|  | MG09955N5 | CCTACCAGGTATGTACTGGTACTTCG |
|  | MG09955R5 | GGCAAAGGAATAGAGTAGATGCCGACCGCTCGAACCTTATCATTGTGGACTCC |
|  | MG09955R3 | CATCGGATGTACGGCTTCTACGTC |
|  | MG09955N3 | CTACGTCGCATCCTTGCGAGTAG |
| MGG_17372 | MG17372L5 | GTGCTCATTCTAGCATTCGGTTCGG |
|  | MG17372L3 | GTTGACCTCCACTAGCTCCAGCCAAGCGTCAAGAGCATCGATCAAAGAAGGGC |
|  | MG17372R5 | GGCAAAGGAATAGAGTAGATGCCGACCGCAGCAGTAGATGGCTTGACAGGAAG |
|  | MG17372R3 | GATCACAGGCATTGAGCATTCCC |
|  | MG17372N5 | CAGGAGCGTGTGTCGTTCCTCAG |
|  | MG17372N3 | CCAAGCAATGAAGAATGGGAGTCC |
| MGG_07997 | MG07997L5 | GTTCGTGAGTGCTTGGTTCTTTGCC |
|  | MG07997L3 | GTTGACCTCCACTAGCTCCAGCCAAGCCGCTGAGTGATGTAGGTTCGATCTG |
|  | MG07997R5 | GGCAAAGGAATAGAGTAGATGCCGACCGCCTATTCTGGCTGTAACCATCAGTCC |
|  | MG07997R3 | CACAGAAAGCTTGAGAGGAGTTGCC |
|  | MG07997N5 | GAATCTCAAGCCCGTCATCACTGC |
|  | MG07997N3 | CTGATGACACTATCCAAGGCAAGGC |
