# Supplementary material for: Transcriptional Divergence Underpinning Sexual Development in the Fungal Class Sordariomycetes
Source: mBio. 2022 May 31;13(3):e01100-22. doi: 10.1128/mbio.01100-22 (PMC9239162; doi:10.1128/mbio.01100-22)
Supplement: FIG S4 [file mbio.01100-22-s0005.pdf]

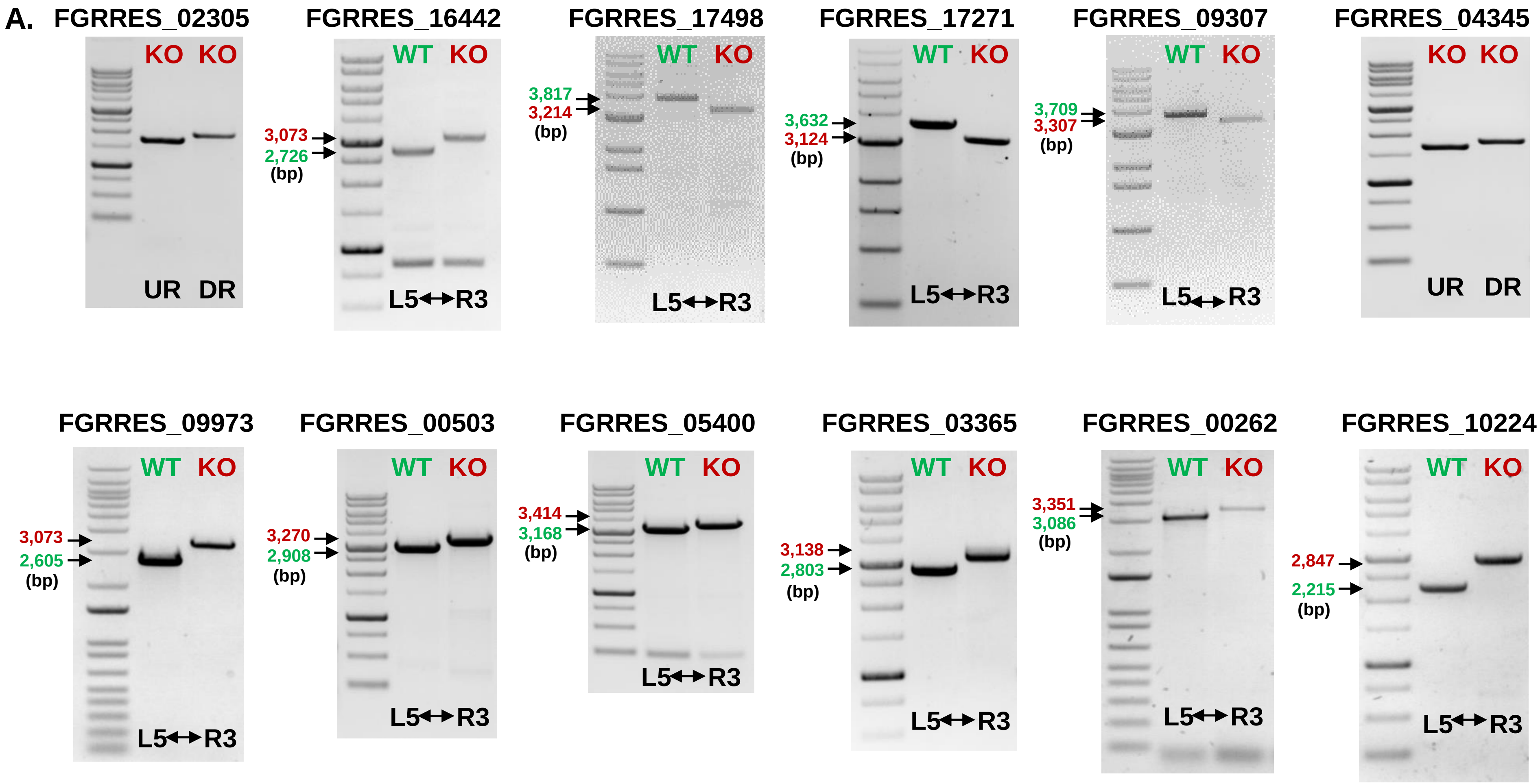

Fig. S4. Confirmation of the gene deletions by PCR. (A-C) The authenticity of knockout mutants was checked in PCR analysis along with their wild-type progenitor (WT; PH-1 strain for *Fusarium graminearum*; 4091, 4091-5-8 strain and 4139, 4136-4-3 strain for *Magnaporthe oryzae*). The primer pairs (see Table S2) used for PCR amplification are indicated below the gels: L5↔R3, L5 and R3 primers were used to distinguish knockouts from the WT progenitor. The expected sizes of PCR amplicons for WT and knockout mutant (KO) were depicted by arrows; the upstream region (UR) and downstream region (DR) of target genes in KOs were amplified, using L5 and HY-R primer pairs and R3 and YG-F primer pairs, respectively, which amplify left and right flanking regions of the target gene and selection marker (here specifically, hygromycin B phosphotransferase gene) that are present only in knockout mutants. (**D**) A schematic diagram indicating the positions of primer pairs; L5↔R3, UR, and DR.

B.

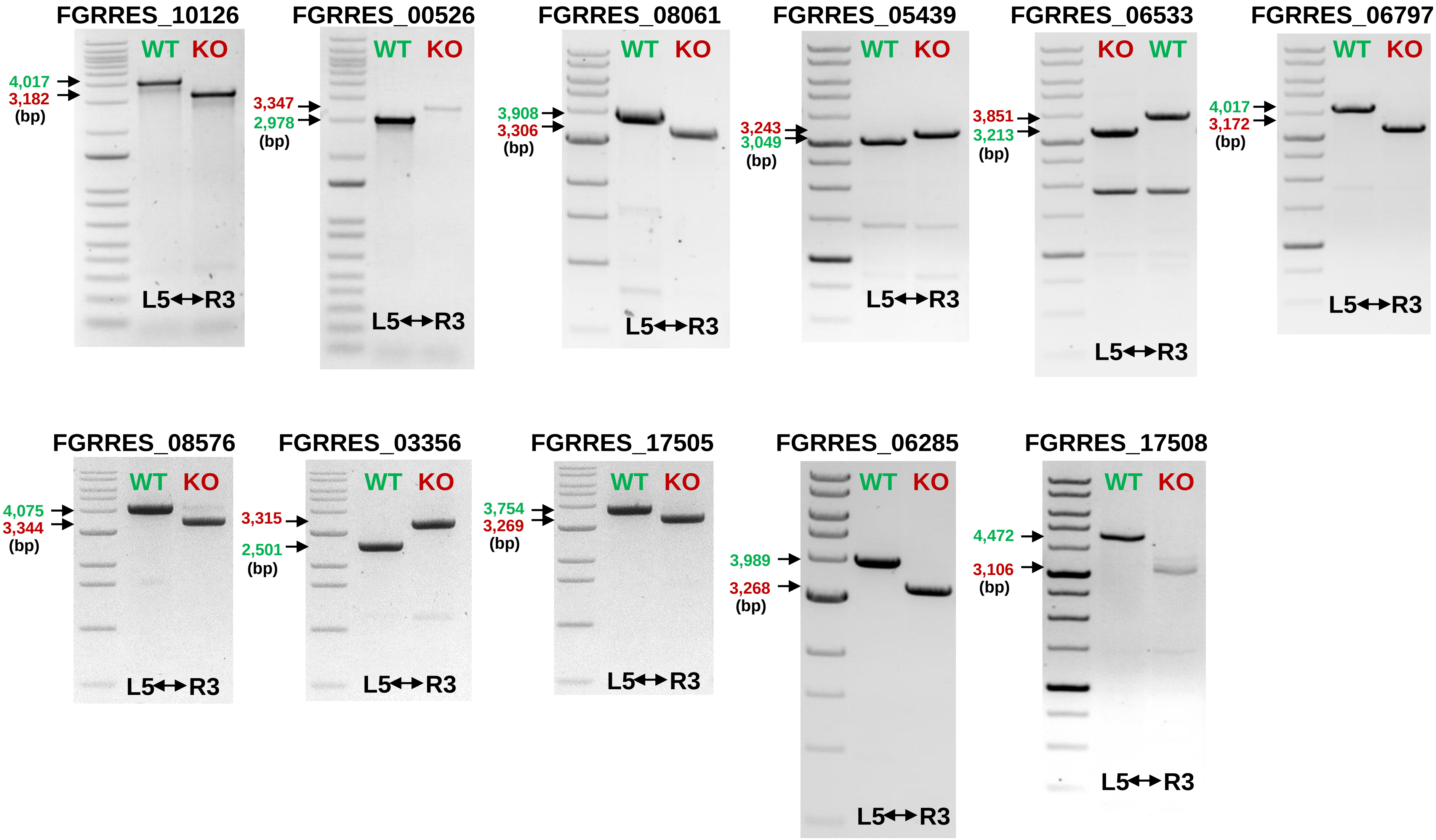

Fig. S4. Confirmation of the gene deletions by PCR. (*continued*)

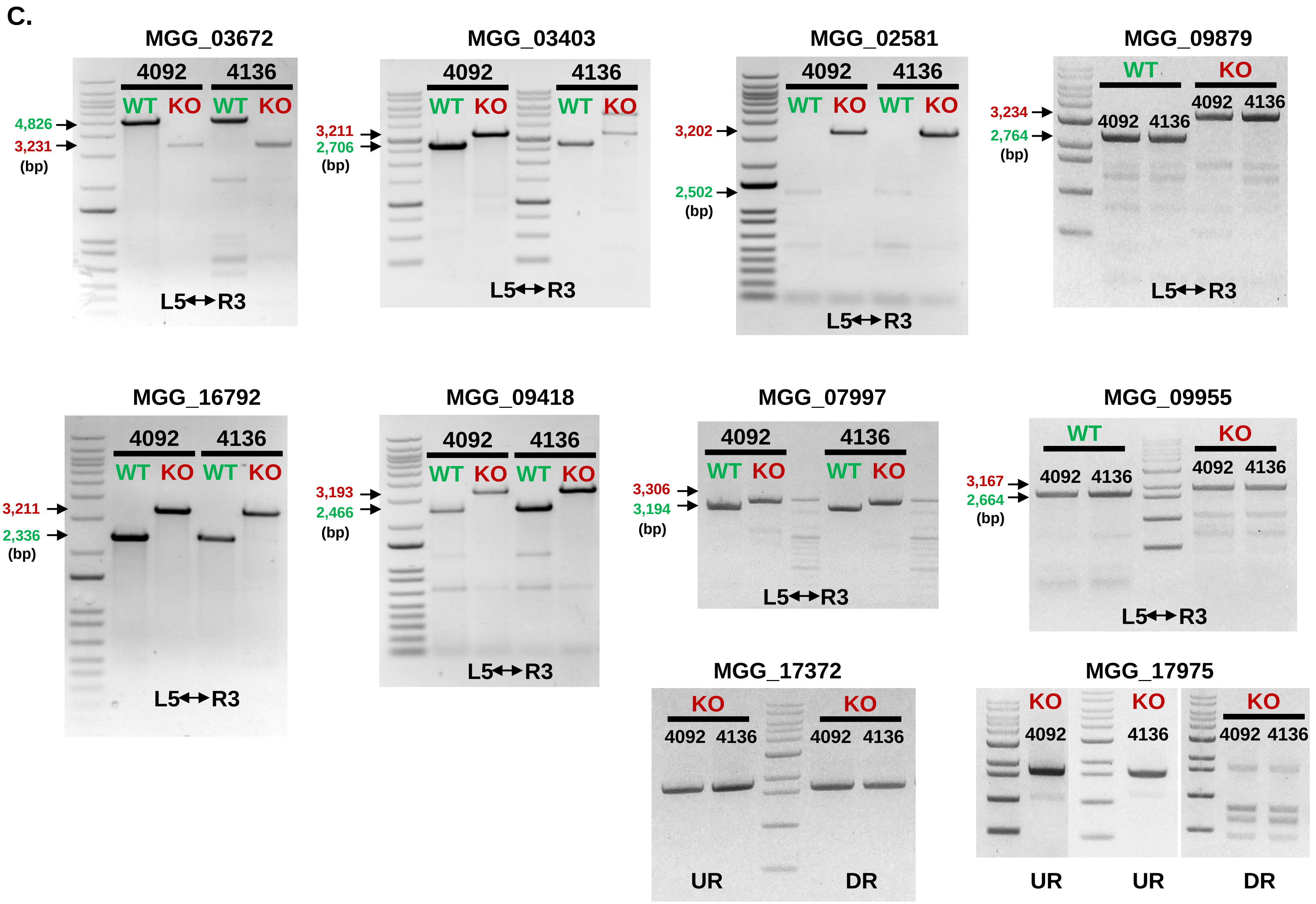

Fig. S4. Confirmation of the gene deletions by PCR. (*continued*)

D.

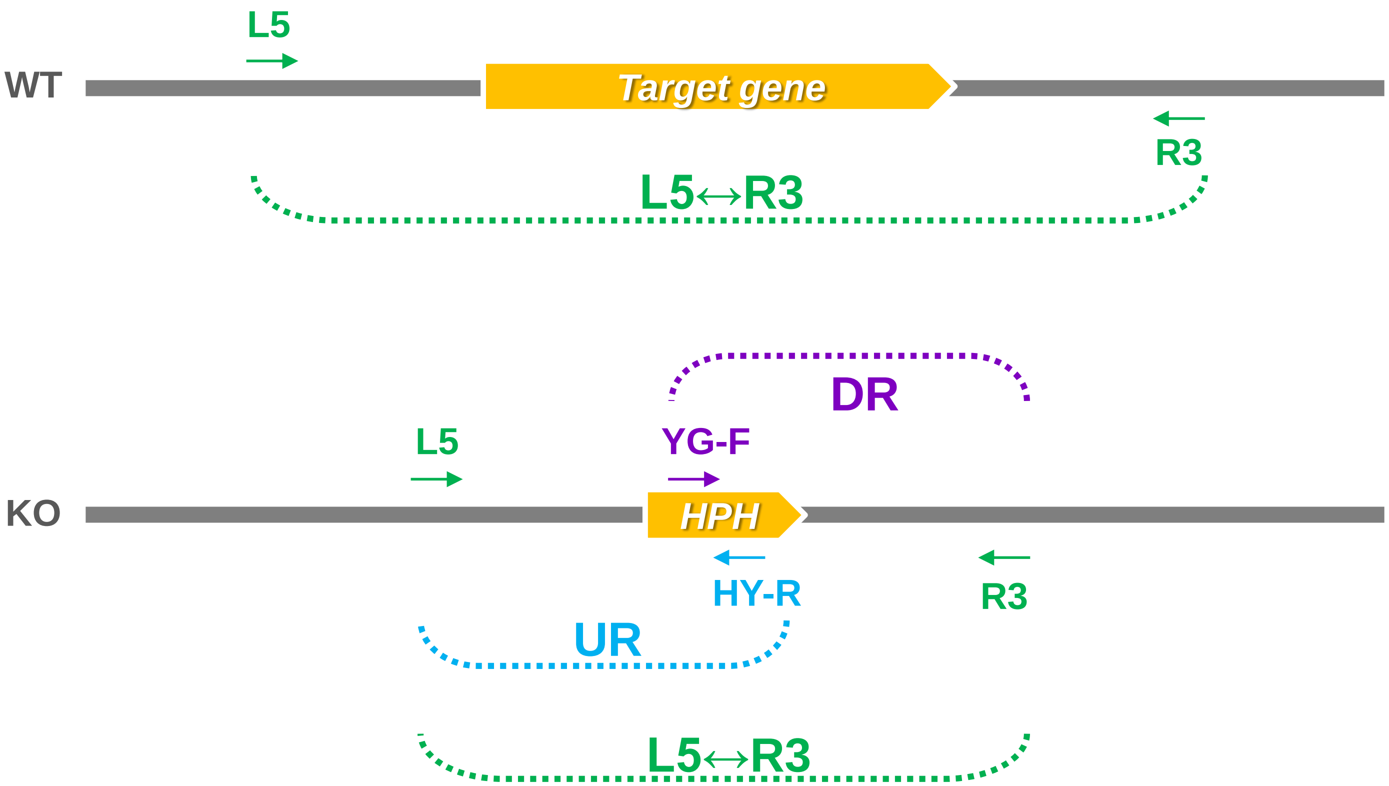

Fig. S4. Confirmation of the gene deletions by PCR. (*continued*)
