# Supplementary material for: Transcriptional Divergence Underpinning Sexual Development in the Fungal Class Sordariomycetes
Source: mBio. 2022 May 31;13(3):e01100-22. doi: 10.1128/mbio.01100-22 (PMC9239162; doi:10.1128/mbio.01100-22)
Supplement: FIG S5 [file mbio.01100-22-s0006.pdf]

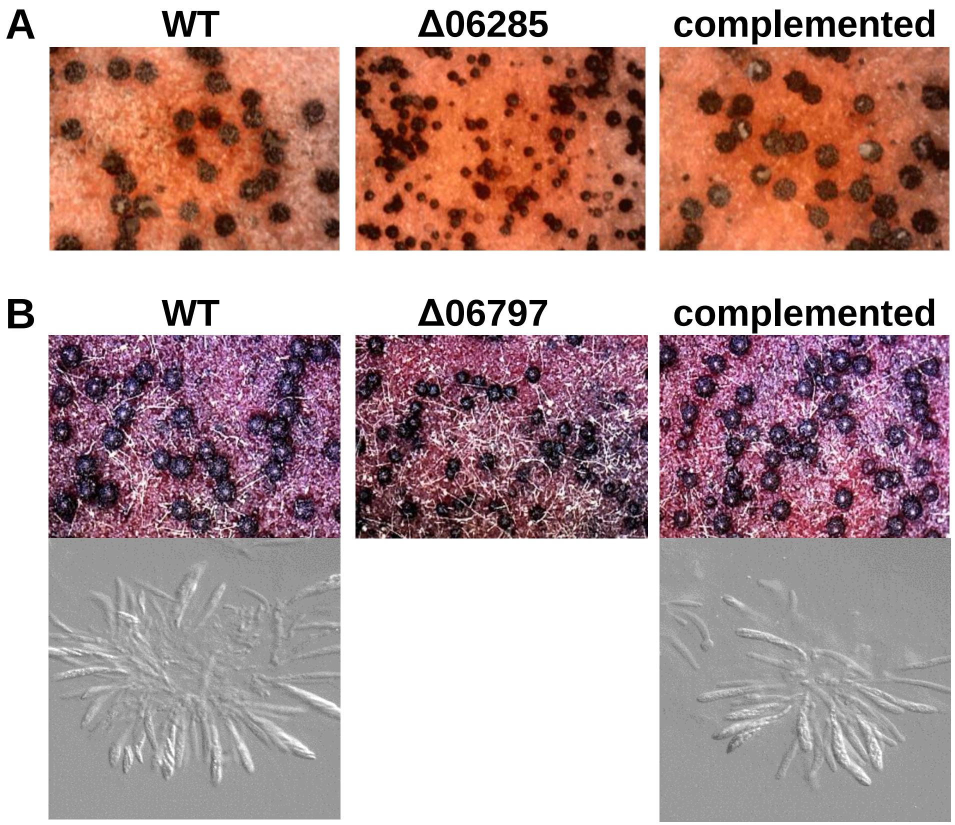

Fig. S5. Genetic complementation of knockout mutants arrested at a protoperithecial stage. (**A**) Perithecia (black spheres) formed on carrot agar. Photos taken 14 days after sexual induction. The wild-type strain (WT) and a complemented strain exude cirrhi, indicative of ascospore production. No cirrhi were produced in knockout mutants of FGRRES_06285. Photos taken 14 days after sexual induction. (**B**) Normal perithecia and ascospore production in the WT and a complemented strain, indicating successful genetic transformation of FGRRES_06797. Photos taken 7 days after sexual induction.
